# Machine learning integration with multi-omics data constructs a robust prognostic model and identifies PTGES3 as a therapeutic target for precision oncology in lung adenocarcinoma

**DOI:** 10.3389/fimmu.2025.1651270

**Published:** 2025-09-29

**Authors:** Lian-jie Ruan, Kang-Qiang Weng, Wei-yu Zhang, Yao-ning Zhuang, Jing Li, Li-ming Lin, Yu-tong Chen, Yi-ming Zeng

**Affiliations:** ^1^ Department of Pneumology, The Second Affiliated Hospital of Fujian Medical University, Quanzhou, Fujian, China; ^2^ Department of Respiratory and Critical Care Medicine, The Affiliated Hospital of Putian University, Putian, Fujian, China; ^3^ Department of Urology, The Affiliated Hospital of Putian University, Putian, China; ^4^ Center for Vascular Surgery and Interventional Oncology, Fujian Provincial Hospital, Fuzhou University Affiliated Provincial Hospital, Fuzhou, China; ^5^ Department of Oncology, Fujian Provincial Hospital, Fuzhou University Affiliated Provincial Hospital, Fuzhou, China; ^6^ Fujian Key Laboratory of Lung Stem Cells, The Second Affiliated Hospital of Fujian Medical University, Quanzhou, Fujian, China

**Keywords:** lung adenocarcinoma, prognostic model, machine learning, PTGES3, ZBTB7A

## Abstract

**Background:**

Lung adenocarcinoma is the most prevalent lung cancer type, with a 5-year survival rate for advanced patients below 20%. This study aims to develop a risk model to guide treatment for these patients.

**Materials and methods:**

RNA-seq data from TCGA and GEO were analyzed using Cox regression and 10 machine learning algorithms to identify prognostic genes and stratify patients. Single-cell datasets were integrated to examine PTGES3’s role in tumor progression, with SCENIC and ATAC-seq revealing its transcriptional regulators. PTGES3 expression was evaluated via tissue microarray immunohistochemistry. Functional assays (CCK-8, colony formation, flow cytometry, Western blot) after lentiviral knockdown in lung cancer cells assessed its effects on proliferation, apoptosis, and cell cycle. ZBTB7A was validated as a transcriptional regulator of PTGES3 by dual-luciferase reporter assay, and xenograft models in nude mice evaluated tumor growth *in vivo*.

**Results:**

Our analysis identified 28 key genes, classifying lung adenocarcinoma samples into high-score and low-score groups. Patients in the high-score group showed worse prognoses, linked to clinical stage progression and phenotypes like angiogenesis and epithelial-mesenchymal transition. PTGES3 knockdown inhibited tumor growth, leading to cell cycle arrest and increased apoptosis. ZBTB7A was identified as a key regulator of PTGES3, while interactions among LGALS9, P4HB, and CD44 significantly impacted signaling pathways influencing the tumor microenvironment’s immune status.

**Conclusions:**

Our findings highlight the potential of LS score-based molecular subtyping to improve treatment strategies for lung adenocarcinoma and emphasize PTGES3’s role in new therapeutic development.

## 1 Introduction

Lung cancer represents the most prevalent type of cancer globally, with lung adenocarcinoma (LUAD) being the most common subtype, accounting for approximately 40% of all lung cancer cases (1). Despite significant advancements in diagnostic and therapeutic modalities, the prognosis for lung cancer patients remains concerning—particularly for those with metastatic LUAD, whose five-year survival rate is less than 20% (2, 3). Moreover, the incidence and mortality rates associated with lung cancer continue to rise, accompanied by increasing challenges related to drug resistance and low overall response rates (4). This situation underscores the urgent need to explore and identify biomarkers that may predict the efficacy of targeted therapies and immunotherapies for LUAD, as well as to investigate their underlying mechanisms (5). Such efforts could provide valuable targets and a theoretical framework for drug design and informed clinical decision-making.

In this study, we aimed to elucidate the relationship between the molecular characteristics of LUAD and patient prognosis. Through a systematic analysis of RNA-seq data from the TCGA and GEO databases, we identified 28 differentially expressed genes that are significantly associated with LUAD prognosis. The selection of these key genes was based on Cox regression analysis combined with the comprehensive application of ten machine learning methods, which ensured the reliability and accuracy of the prognostic markers identified. Building on this foundation, we developed a prognostic scoring system, termed the LS score, which effectively reflects patient survival outcomes. Our analysis of the LS score allowed for the categorization of LUAD patients into high-score and low-score groups. The results demonstrated significant differences in clinical characteristics between these two molecular subtypes. Patients in the high-score group generally faced a more unfavorable prognosis and exhibited strong correlations with various tumor biological behaviors, including angiogenesis, cell cycle regulation, and epithelial-mesenchymal transition. This classification not only aids in the identification of prognostic risk among patients but also serves as a foundation for the development of tailored clinical treatment strategies.

**Figure 1:**
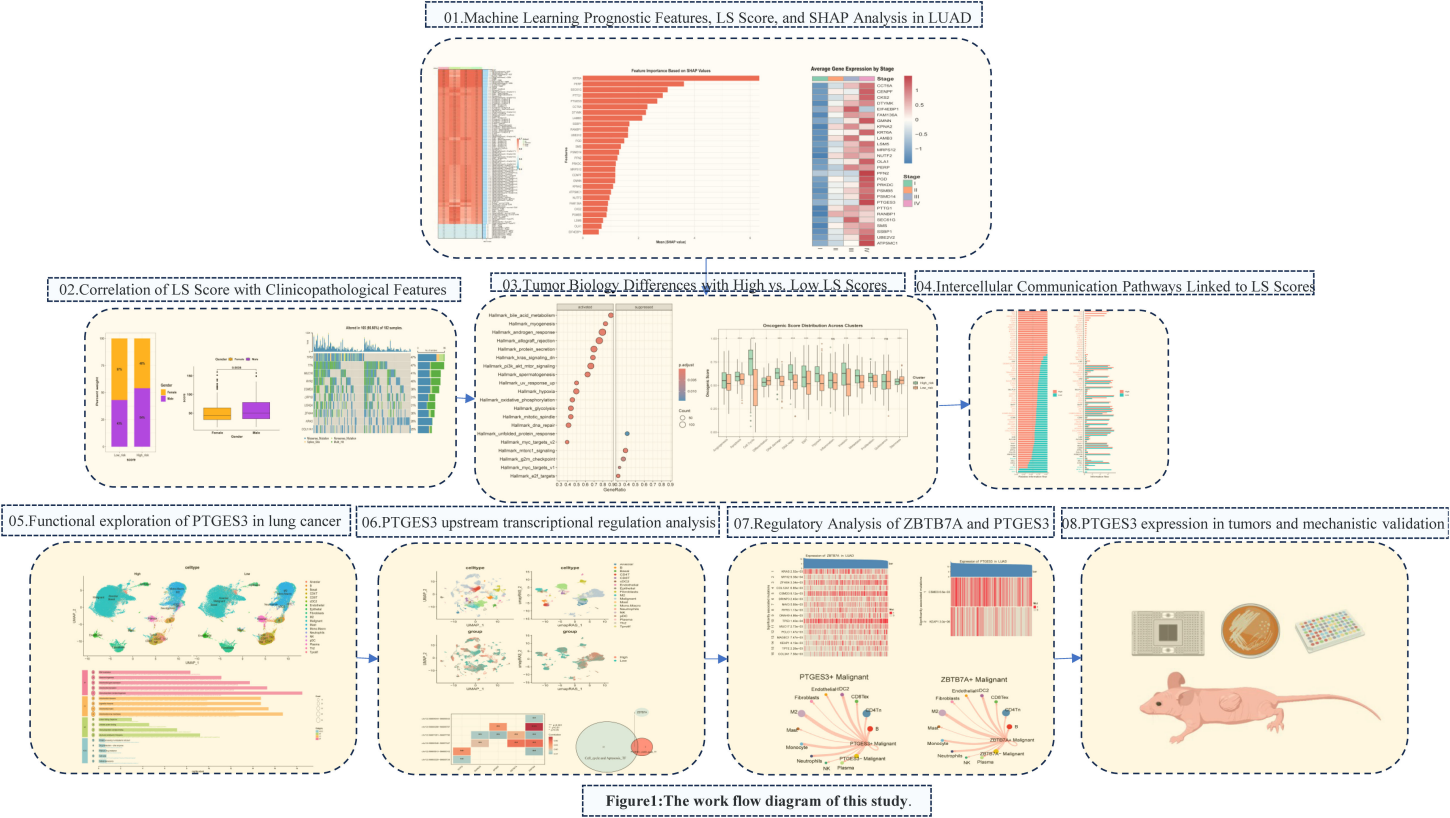
The work flow diagram of this study.

We particularly focused on the key gene PTGES3 within our model, which has been recognized as a prognostic biomarker in multiple cancers, including breast cancer, ovarian cancer, glioblastoma, and liver cancer (6–9). PTGES3 also plays an important role in immune regulation in LUAD (10, 11). Our analyses assessed the role of PTGES3 in tumor progression, demonstrating its relationship with cell cycle and apoptosis, which were further corroborated by both *in vitro* and *in vivo* experiments. Additionally, we investigated the key upstream transcription factor ZBTB7A along with the significant upstream mutations CSMD3 and KEAP1 that influence PTGES3 expression. In terms of cell communication, LGALS9, P4HB, and CD44 may interact within signaling pathways involving the transcription factor ZBTB7A and its target gene PTGES3 in tumor cells and macrophages. This interaction may subsequently impact the immune status of the tumor microenvironment and influence tumor progression. The whole research process is shown in **Figure 1**.

## 2 Materials and methods

### 2.1 Data sources

We obtained data from TCGA-LUAD and GSE42127, which were sourced from the UCSC-XENA and GEO databases, respectively. After applying the “combat” method to mitigate batch effects, these datasets were merged to form a new cohort referred to as the Merge cohort. To further validate the correlation between ZBTB7A and PTGES3, we utilized GSE11969, GSE19188, and GSE72094. Additionally, we accessed single-cell data (GSE117570, GSE127465, GSE148071) from the TISCH database (http://tisch.comp-genomics.org/) to analyze tumor tissue (12), resulting in a dataset comprising 36,096 genes across 111,138 cells. Single-cell analysis was conducted using the Seurat R package, with quality control and cell annotation performed based on the information from the TISCH database. Furthermore, we curated data from the Cancer Cell Atlas database (https://www.weizmann.ac.il/sites/3CA/) to investigate PTGES3 expression across various tumor cell datasets (13).

### 2.2 Machine learning to establish LS score prognostic model

We employed several integrative machine learning algorithms, including Lasso, Ridge, Elastic Net (Enet), Random Forest (RF), Stepwise Generalized Linear Model (Stepglm), Generalized Boosted Regression Modeling (GBM), Support Vector Machine (SVM), Extreme Gradient Boosting (XGBoost), glmBoost, and Naive Bayes. We paired these ten algorithms to create a variety of combinations of machine learning algorithms. The TCGA data served as the training set, while the GSE42127 and merged cohort data were utilized for validation. To construct the prognostic model, the C-index for each model was calculated as the mean across the three datasets. Detailed information on various combinations can be found in **Supplementary Table 1**.

We utilized the R package pROC to generate ROC curves and calculate the area under the curve (AUC) for different models, assessing the predictive strength of genes in disease occurrence. Additionally, we performed survival analysis using the R packages “survival” and “survminer.” To elucidate the selected genes, we applied SHAP (SHapley Additive exPlanations) methods to interpret the model output. SHAP, based on game theory, explains the output of any machine learning model by evaluating the significance of features on output changes when combined with others.

### 2.3 Effect analysis of knockdown genes in the DepMap database

In the DepMap database (14), Gene Effect refers to the impact of interference operations, such as gene knockout or knockdown, on cell viability. A negative Gene Effect indicates that the gene is essential for cell survival; specifically, knockout of this gene adversely affects cell proliferation. Furthermore, a smaller Gene Effect value suggests a greater necessity of that gene, indicating its critical role in cell survival and function, as well as a higher level of dependency by the cell.

We utilized the GSCA database (https://guolab.wchscu.cn/GSCA/#/) to analyze the relationships between the top 10 genes from the prognostic model, immune infiltration, and drug sensitivity (15).

### 2.4 Clinical and molecular features of patients with high and low LS scores

We analyzed the relationship between LS scores calculated by the RSF random forest method and clinical-pathological features. The “Maftools” package was used to analyze gene mutations in high-scoring and low-scoring groups. Additionally, we created heatmaps of chemokines and immune cell-related genes for each scoring group using the “pheatmap” R package. Univariate and multivariate regression analyses were conducted to evaluate the prognostic impact of LS scores.

In terms of molecular characteristics, we performed a correlation analysis between model scores and all genes, presenting the results via heatmaps. Based on the correlation analysis results, we conducted GSEA enrichment analysis using the R package “clusterProfiler.” To assess differences in tumor progression phenotypes, we compared mDNAsi, mRNAsi, and TMB using ssgsea scores. Furthermore, we collected signatures related to tumor stemness and utilized the CancerSEA database (http://biocc.hrbmu.edu.cn/CancerSEA) to categorize 14 distinct functional states of tumor cells, allowing us to compare the differences in tumor progression phenotypes between high and low scoring groups (16).

### 2.5 Expression and biological function of key gene PTGES3 in lung cancer

We utilized the Curated Cancer Cell Atlas database to analyze the expression of PTGES3 across various tumors using single-cell datasets. The single-cell data from GSE117570, GSE127465, and GSE148071 were downloaded from the TISCH database, focusing on tumor tissue data. For single-cell analysis, we employed the R package Seurat, using quality control and cell annotation informed by the TISCH database results. We identified differentially expressed genes among various cell types using the FindAllMarkers function, showcasing the top 5 genes with high and low expression for each cell type.

Based on the median expression level of the PTGES3 gene, all cells were categorized into high and low expression groups, and UMAP plots were generated to illustrate the grouping results. Additionally, we conducted trajectory analysis using the R package Slingshot to investigate the relationship between gene expression and pseudotime. Enrichment analysis was performed to further compare the participation of high and low PTGES3 expression groups in various biological processes.

### 2.6 Cell communication analysis

To further investigate the differences in cell communication between high and low PTGES3 groups, we employed the R package CellChat to analyze the information flow between these groups. Additionally, we examined the tumor-cell interactions involving PTGES3 and ZBTB7A using CellChat.

### 2.7 PTGES3 transcriptional regulation analysis

This study utilized the SCENIC method, which includes co-expression analysis using GENIE3 to construct a co-expression network, motif enrichment analysis via RcisTarget to establish a regulon model, and activity evaluation through AUCell to assess regulon activity in cells (17). This approach integrates cis-regulatory sequence data with RNA sequencing, yielding a list of regulons (transcription factors and target genes) and regulon activity scores (RAS). The analysis helped identify upstream transcription factors associated with PTGES3, which is linked to the cell cycle and apoptosis.

The annotatePeak function from the ChIPseeker package annotated the PTGES3 promoter’s transcription start site (TSS) within the region tssRegion=c (–3000, 3000). The LUAD_ATAC dataset is available from the NCI Genomic Data Commons(NCI Genomic Data Commons).

### 2.8 ZBTB7A transcription factor analysis upstream of PTGES3

The SCENIC method was employed to identify key regulatory transcription factors, followed by intersection analysis with ATAC data. In the NSCLC_GSE125465 single-cell dataset, the FeaturePlot function from the Seurat package was utilized to visualize the colocalization and correlation between ZBTB7A and PTGES3 in tumor cells. Additionally, the interactions between ZBTB7A-positive tumor subpopulations and other cell subsets were further examined using CellChat, and the transcriptional activity of ZBTB7A in different cell subsets with high and low PTGES3 expression was compared. Furthermore, Sangerbox (http://www.sangerbox.com/tool) was used to analyze the expression levels of ZBTB7A across various cancers (18).

### 2.9 Comparative analysis of genetic mutations in PTGES3 and ZBTB7A

We utilized the online database Sparkle (https://grswsci.top) to investigate the relationship between gene expression and specific gene mutation types. To achieve this, we employed the coin package in R and its independence_test function to conduct permutation tests, thereby validating this association. Patients were classified into mutant and wild-type groups based on their gene mutation status, and differential analysis was performed using the limma package to calculate the log2 fold change (log2FC) for each gene. We then ranked the genes according to their log2FC values. Subsequently, we conducted gene set enrichment analysis using the clusterProfiler package, focusing on both the Hallmark gene set and the KEGG metabolic gene set. The enrichment score (ES) for each gene set was calculated, followed by significance testing and multiple hypothesis testing of the ES values. Finally, we performed visualizations that highlighted pathways with an adjusted p-value of less than 0.05.

### 2.10 Materials and reagents

The antibodies used in this study were as follows: anti-PTGES3 (1:2000, Signalway Antibody, #32773), anti-Bcl-2 (1:2000, Proteintech, #12789-1-AP), anti-Bax (1:2000, Proteintech, #50599-2-Ig), anti-Ki67 (1:5000, Proteintech, #27309-1-AP), anti-GPX4 (1:2000, Proteintech, #67763-1-Ig).Additionally, antibodies against CDK4 (1:1000, Cell Signaling Technology, #12790), Cyclin D1 (1:1000, Cell Signaling Technology, #2978), and GAPDH (1:5000, Cell Signaling Technology, #2118) were utilized. Fetal bovine serum (FBS), Trypsin-EDTA (0.25%) solution, Pierce™ BCA Protein Assay kit, RPMI 1640 medium, and FxCycle PI/RNase Staining Solution were purchased from Thermo Fisher Scientific (Waltham, MA, USA). Annexin V Apoptosis Detection kit and CCK-8 kit were obtained from Abbkine (Wuhan, Hubei, China).

### 2.11 Immunohistochemistry based tissue microarray

Tissue microarrays (TMAs) containing lung cancer and adjacent non-cancerous tissues (Cat: HLugA180Su08) were obtained from Shanghai Outdo Biotech Company (Shanghai, China). The usage of this commercial TMA was approved by the Institutional Review Board of Shanghai Outdo Biotech Company (No.SHYJS-CP-1904014), and the study was conducted in accordance with the ethical principles of the Declaration of Helsinki. The ethical approval documents are provided in **Supplementary Table 2**. IHC was conducted to assess PTGES3 expression (dilution 1:4000). Scoring was performed by two experienced pathologists, blinded to tissue identity, utilizing a grading system based on staining intensity (0: none; 1: weak; 2: moderate; 3: strong) and the percentage of positive-staining cells (1: 1-25%; 2: 26-50%; 3: 51-75%; 4: 76-100%). The final score was calculated as intensity score multiplied by percentage score. The clinicopathological features of lung cancer patients are summarized in **Table 1**.

**Table 1:** Clinicopathological features of 80 lung adenocarcinoma patients in tissue microarray.

Characteristics		N (%)
Age (years)	<65	53 (66)
	≥65	27 (34)
Sex	Females	31 (39)
	Males	49 (61)
Tumor size	≤5 cm	60 (75)
	>5 cm	20 (25)
Clinical stage	I	2 (2.5)
	II	47 (58.8)
	III	31 (38.7)
T stage	T1	19 (23.8)
	T2	43 (53.7)
	T3	15 (18.7)
	T4	3 (3.8)
N stage	N0	40 (50)
	N1	15 (18.7)
	N2	11 (13.8)
	N3	14 (17.5)
M stage	M0	79 (98.8)
	M1	1 (1.2)
Lymph node metastasis		8 (10)
Distant metastasis		0 (0)

### 2.12 Cell lines and cell culture

The lung cancer cell lines (H1299 and A549) were obtained from the Cell Bank of the Chinese Academy of Sciences (Shanghai, China). H1299 and A549 cells were cultured in RPMI 1640 medium, which was supplemented with 10% fetal bovine serum, 100 U/mL penicillin, and 100 μg/mL streptomycin (Hyclone, Logan, UT, USA) at 37 °C in a humidified atmosphere of 5% CO2.

### 2.13 Lentivirus transfection

Specific short hairpin RNAs (shRNAs) targeting PTGES3 and a non-silencing control shRNA were designed and validated by OBiO Technology (Shanghai, China). The shRNA sequences were: sh-PTGES3-1: GGCTTAGTGTCGACTTCAATA; sh-PTGES3-2: GAAGACAGTAAGGATGTTAAT; sh-PTGES3-3: CGAAGGGACTATGTCTTCATT; sh-Ctrl: CCTAAGGTTAAGTCGCCCTCG. Lentiviral particles containing these shRNAs were transfected into H1299 and A549 cells at a multiplicity of infection (MOI) of 10 for 6–8 hours. Following transfection, the medium was replaced with fresh culture medium and the cells were co-cultured for an additional 72 hours. Stable cell lines were then selected using puromycin and used for subsequent experiments.

### 2.14 Western blot analysis

Western blot analysis was performed as described in our previous publication (19). Proteins were extracted using standard procedures, and concentrations were measured with a BCA Protein Assay Kit (Beyotime, Shanghai, China). Equal amounts of protein were loaded onto a 10% gel for electrophoresis and transferred to a PVDF membrane. After blocking, membranes were incubated with primary (1:1000) and secondary antibodies (1:5000), and protein bands were detected using an ECL imager (Bio-Rad, Hercules, CA, USA). All procedures followed our established protocols.

### 2.15 Cell viability assay

Cells were transfected and were reseeded into 96-well plates at a density of 0.2×10–5 cells/well and cultured for 24, 48, 72, 96, 120 hours and 10 µL of CCK-8 solution was added to each well and was incubated at 37 ˚C for 2 hours in an incubator with 5% CO2 in the dark. Subsequently, the absorbance was measured at 450 nm using a microplate reader (Thermo Fisher Scientific; Carlsbad, CA, USA).

### 2.16 Colony formation assay

Cells were transfected and reseeded into 12-well plates at a density of 500 cells/well and cultured at 37°C in an atmosphere of 5% CO2 for 8–10 days. At the end of the experiment, the cells were fixed with 4% paraformaldehyde for 15 min and then stained with 0.1% crystal violet for 15–20 min, followed by washing with PBS. The number of colonies was counted.

### 2.17 Cell cycle and apoptosis assay

Cells were transfected and reseeded into 6-well plates at a density of 1.0 × 10^5 cells/well, then cultured for 72 hours. For the cell cycle assay, cells were collected, fixed with 70% ethanol at 4°C overnight, centrifuged at 2000 rpm for 3 min, washed, and incubated with FxCycle PI/RNase Staining Solution for 30 min at room temperature. Cell cycle progression was analyzed by FACS (Becton Dickinson, CA, USA) using Modfit LT v3.0 (Verity Software House). For the apoptosis assay, cells were washed with PBS and incubated with an Annexin-V Apoptosis Detection Kit for 15 min, and the apoptotic rate was analyzed via FACS.

### 2.18 Dual luciferase reporter assay

We obtained the overexpression ZBTB7A plasmids and the PTGES3 mutant and wild-type plasmids from Miao-Ling Plasmid (China) and transfected them following the manufacturer’s instructions. The reagents for the dual luciferase reporter assay were purchased from Yeasen (Shanghai, China), and the transfection and detection were carried out according to the provided protocols.

### 2.19 RNA sequencing

Stable knockdown transformants were sent to CapitalBio Technology for transcriptome sequencing. RNA was extracted using TRIzol, and the concentration was measured with a Nanodrop ND-2000, with integrity assessed using an Agilent Bioanalyzer 4150, yielding values between 9.1 and 9.6. Sequencing libraries were prepared following the ABclonal mRNA-seq Lib Prep Kit protocol. The purified cDNA libraries were sequenced on the MGISEQ-T7 platform. KEGG analyses identified enriched pathways and altered biological processes resulting from the knockdown.

### 2.20 Xenograft experiments in nude mice

Male BALB/c nude mice (6–8 weeks, 20 ± 2 g) were obtained from Shanghai SLAC Laboratory Animal Co. Ltd. and housed at Fujian University of Traditional Chinese Medicine. All animal procedures complied with ARRIVE guidelines and the National Research Council’s Guide for the Care and Use of Laboratory Animals. The experimental protocol was approved by the Animal Care and Use Committee of Fujian University of Traditional Chinese Medicine (approval no. FJTCM IACUC 2022174). H1299 cells were transfected with sh-Ctrl and sh-PTGES3, then injected subcutaneously (1×10^6 cells in 100 µL PBS with 50% Matrigel) into the flanks of nude mice (n=6). Tumor volume was measured every other day with a vernier caliper using the formula: (larger diameter × smaller diameter²)/2.

### 2.21 Statistical analysis

Data analyses and presentations were conducted using R software (version 4.3.1) and Python (version 3.10.11). Results are expressed as mean ± standard deviation. Student’s t-test or Mann-Whitney U test was used for two-group comparisons, while one-way ANOVA or Kruskal-Wallis H test was used for multiple group comparisons. Kaplan-Meier survival differences were assessed with the log-rank test. Statistical significance was defined as P < 0.05. Part of the flow chart was drawn on the online Platform GDP (BioGDP - Generic Diagramming Platform for Biomedical Graphics) (20).

## 3 Results

### 3.1 To construct a prognostic gene signature for LUAD patients

**Figure 2:**
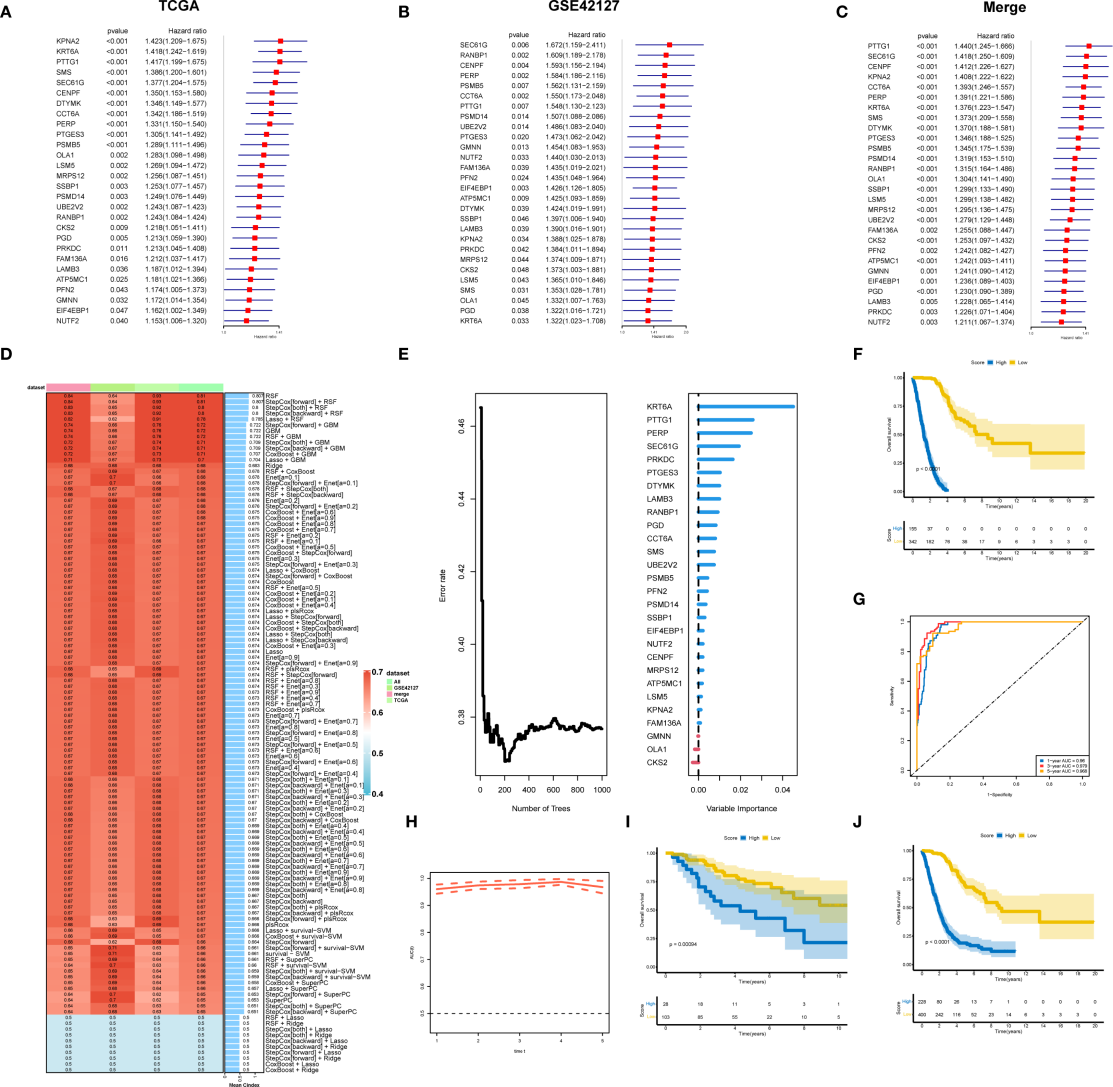
A prognostic gene signature for LUAD patients. **(A)** Results of univariate prognostic regression analysis of TCGA-LUAD. **(B)** Results of univariate prognostic regression analysis of GSE42127. **(C)** Univariate prognostic regression analysis results combining TCGA-LUAD and GSE42127. **(D)** TCGA data were used as the training set, with other datasets as validation sets. Ten machine learning algorithms and various combinations were employed to construct prognostic models, with the obtained C-indexes displayed. **(E)** Twenty-eight genes were modeled using RSF, highlighting their importance. **(F)** Survival analysis for the training dataset TCGA-LUAD. **(G)** Evaluation of the random forest model’s prognostic prediction capability in the training set using the ROC curve. **(H)** Time-dependent ROC curve. **(I)** Survival analysis of the GSE42127 validation set. **(J)** Survival analysis of the merged cohort validation set.

To construct a prognostic gene signature for LUAD patients, TCGA_LUAD and GSE42127 were used for differential gene analysis, and the merged cohort was formed. Survival analysis was performed for the differentially expressed genes, and the results were presented as univariate regression forest plots of TCGA_LUAD prognosis (**Figure 2A**) and GSE42127 prognosis (**Figure 2B**), respectively. By intersection analysis of the COX regression results of the two datasets, genes that were either risk genes or protective genes in the two datasets were selected, resulting in the identification of 28 risk genes and 0 protective genes for machine learning modeling (**Figure 2C**).

We used an integrated program based on machine learning to develop a prognostic LS score using the expression profiles of 28 differential genes related to prognosis. The TCGA dataset was used as the training set, while the remaining datasets served as the validation set. We employed ten machine learning algorithms and their combinations to construct prognostic models, calculating the C-indexes (**Figure 2D**). The results showed that the RSF (random survival forest) algorithm had the best performance. We then modeled the RSF for these 28 genes and illustrated the importance of the genes (**Figure 2E** **).** Survival analysis in the training set TCGA-LUAD (**Figure 2F**) demonstrated that patients with higher LS scores had a worse prognosis. Additionally, the ROC curve was utilized to evaluate the prognostic prediction ability of the random forest model, with results indicating that the AUC value exceeded 0.9 (**Figures 2G, H**), confirming good predictive performance. Prognostic analysis in the validation set GSE42127 and the merged cohorts further validated that patients with higher LS scores had a worse prognosis (**Figures 2I, J**).

### 3.2 RSF model interpretation

**Figure 3:**
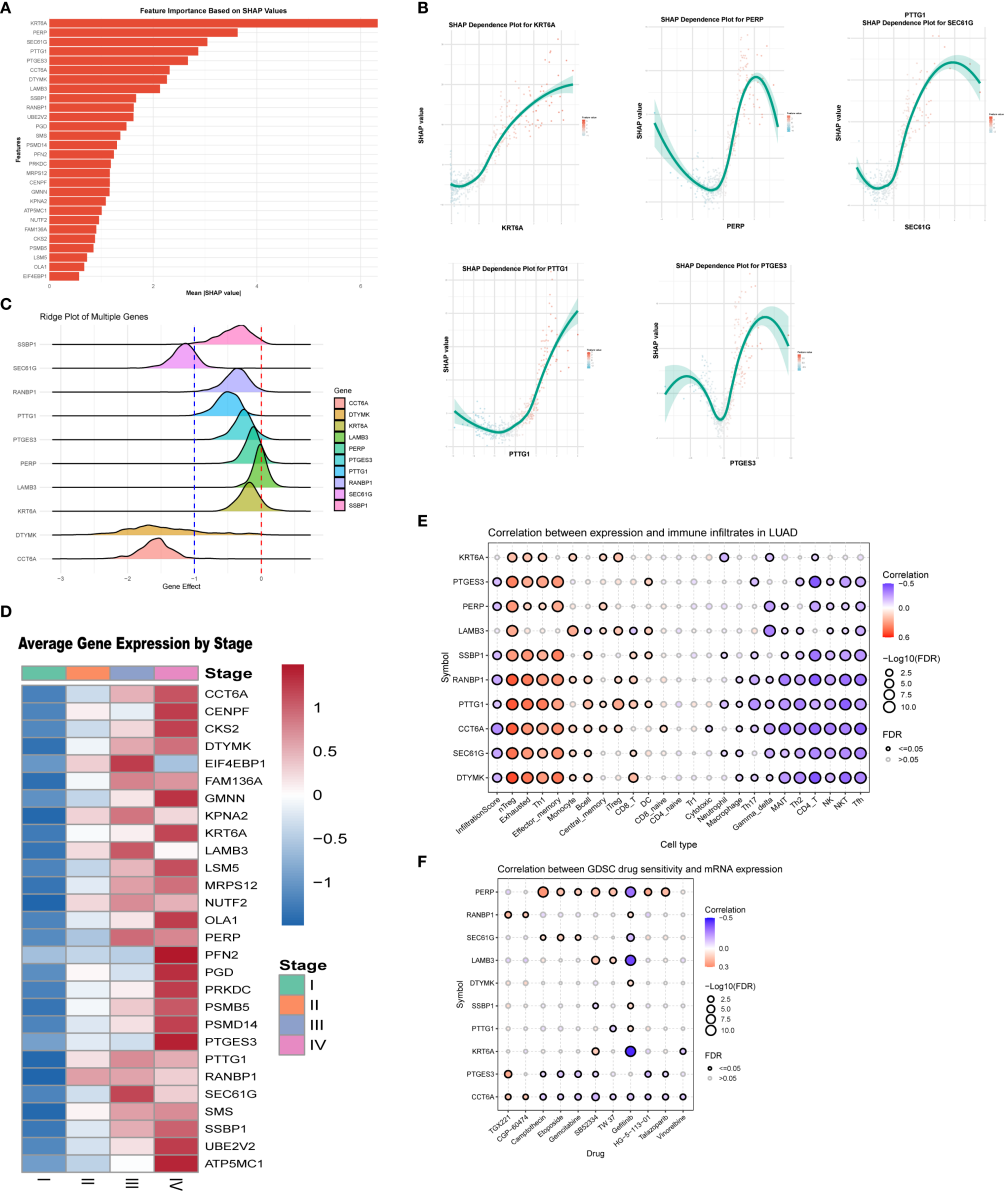
Key gene analysis of the LS model. **(A)** SHAP analysis of the RSF model revealed the distribution of SHAP values for the featured genes, highlighting the varying impacts of different genes on model predictions. **(B)** The SHAP dependence plot illustrates the relationship between feature values and SHAP values, identifying the top five genes that exert the greatest influence on predictions. **(C)** The Cancer Dependency Map (DepMap) analysis assessed the effects of gene knockdown on cell viability and functionality. **(D)** Results indicate that the expression of key genes increases with tumor progression. **(E)** We also analyzed the association between the top ten genes and immune cell infiltration in lung adenocarcinoma. **(F)** Finally, the relationship between gene expression levels and IC50 values of anticancer drugs was assessed, revealing a positive correlation that suggests high gene expression may contribute to resistance against these drugs.

SHAP analysis was conducted on the RSF model, revealing the distribution of SHAP values for key characteristic genes (**Figure 3A**), which underscores the influence of different genes on model predictions. The top five influential genes identified were KRT6A, PERP, SEC61G, PTTG1, and PTGES3. The relationship between the feature values of these genes and their corresponding SHAP values is illustrated in the SHAP dependence plot (**Figure 3B**). Higher SHAP values correlate with an increased probability of poor prognosis, with KRT6A demonstrating the most significant impact; its expression level positively influences poor prognosis likelihood. While PTGES3 appears to exert a protective effect at lower expression levels, increased expression of PTGES3 is associated with a greater negative impact on prognosis.

Using DepMap data analysis, we evaluated the effects of gene knockdown on cell viability using the “Gene Effect” metric. A negative Gene Effect value typically indicates that a gene is essential for cell survival, with smaller negative values reflecting a greater importance for cell viability. The ridge plot (**Figure 3C**) illustrates the distribution of effects for key genes, the majority of which show negative values, indicating a strong association with tumor growth. Positions further to the left on the plot correlate with a more pronounced inhibitory effect on cell proliferation following gene knockout. Interestingly, the expression levels of these key genes increased with stage progression (**Figure 3D**).

In analyzing the relationships between these key genes, immune cell infiltration, and drug sensitivity, we found that high expression of KRT6A was positively correlated with the infiltration of specific immune cell types. Conversely, PTGES3 exhibited complex correlations across various immune cell infiltrates, showing positive correlations with effector and exhausted T cells, while demonstrating negative correlations with CD4+ T cells and NK cells (**Figure 3E**). Drug sensitivity analysis indicated that high KRT6A expression may correlate with sensitivity to gefitinib, whereas PTGES3 may confer resistance to TGX221 (**Figure 3F**).

### 3.3 Correlation between LS score and clinicopathological features of LUAD patients

**Figure 4:**
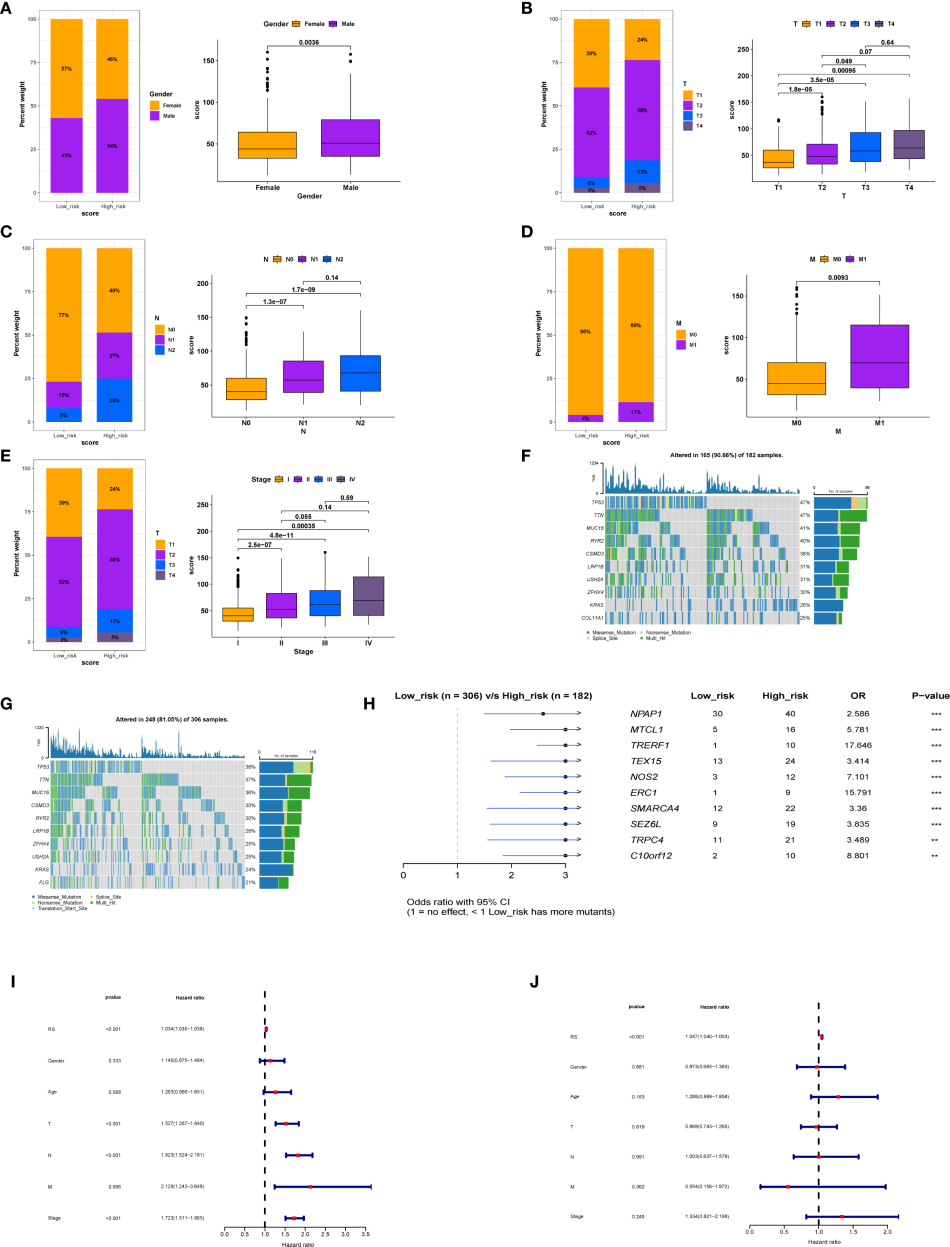
Relationship between model score and clinical factors in patients. **(A)** Association between model score and gender. **(B)** Correlation between model score and T stage. **(C)** Relationship between model score and N stage. **(D)** Relationship between model score and M stage. **(E)** Association between model score and overall stage. **(F)** Gene mutation status in the high-score group. **(G)** Differences in gene mutation profiles between the low-score group. **(H)** Comparative analysis of gene mutations between high and low-score groups. **(I)** Univariate analysis indicating that model score is an independent prognostic factor. **(J)** Multivariate regression analysis confirming that model score serves as an independent prognostic factor.

Comparative analyses with respect to variables such as sex, T stage, N stage, M stage, and clinical stage showed significant associations between the low-score group and the high-score group (**Figures 4A-E**). In addition, when we compared the rates of somatic mutations in significantly mutated genes (SMGs) according to score group, mutations in TP53 (47% vs. 38%), TTN (47% vs. 37%), and MUC16 (41% vs. 36%) were more prevalent in the high-score group (**Figures 4F, G**).

Further examination of the differentially expressed genes between the low-score group and the high-score group (**Figure 4H**) showed that these genes may be involved in the regulation of tumor cell growth, migration, metastasis, and drug resistance. The significantly differentially expressed genes included NPAP1, MTCL1, TRERF1, and TEX15. Univariate and multivariate regression analyses of the LS score suggested that it was an independent predictor of prognosis (**Figures 4I, J**).

### 3.4 Differences in tumor biology between patients with high and low scores

**Figure 5:**
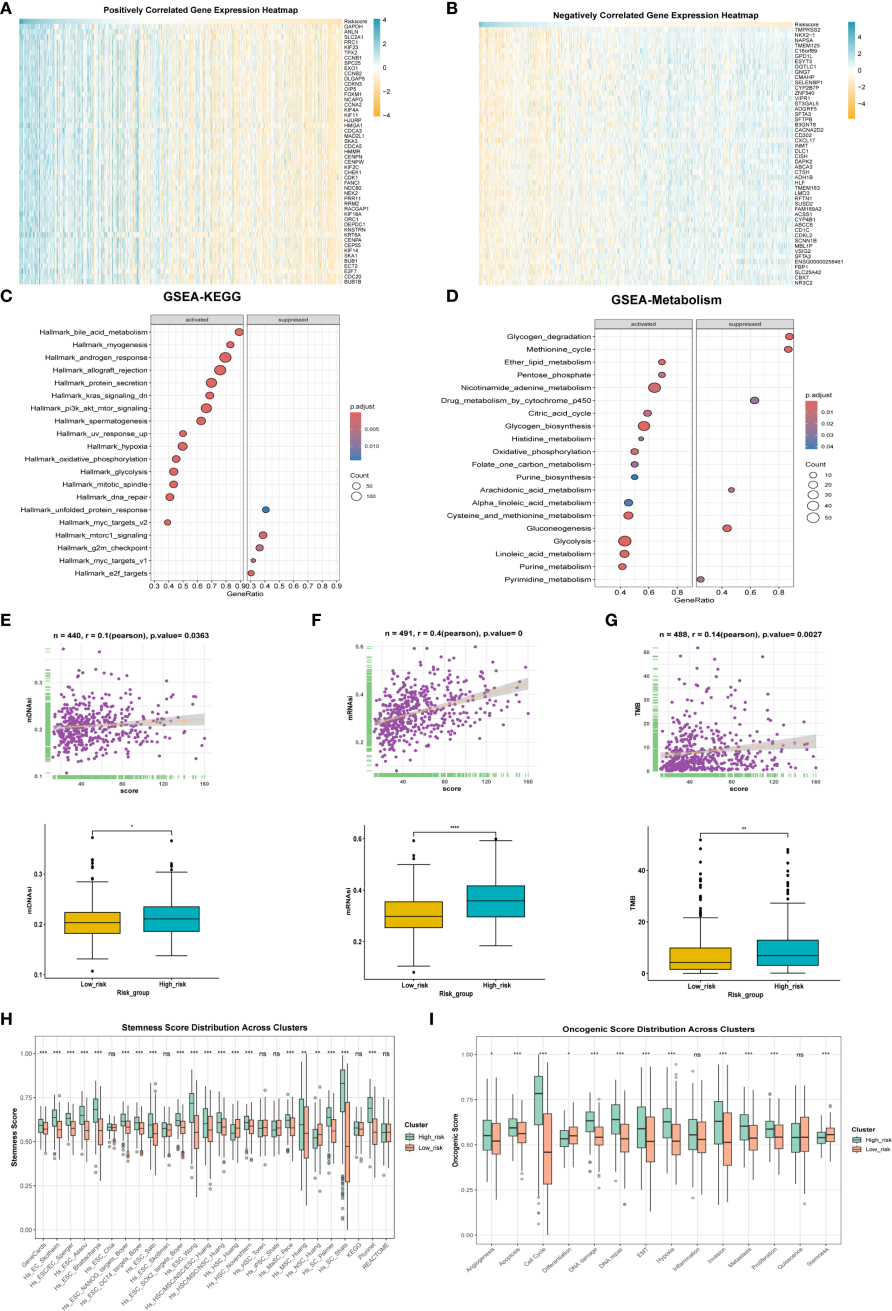
Differences in tumor biology between patients with high and low scores. **(A)** The correlation analysis between the model score and all genes, respectively, showed the positively correlated top50 genes using heat maps. **(B)** Expression levels of inversely correlated top 50 genes. **(C)** GESA enrichment analysis was employed to examine the differences in enriched pathways between high and low score groups. **(D)** GESA enrichment analysis was utilized to compare the metabolic enrichment differences between high and low score groups. **(E)** mDNAsi expression differences and correlation between high and low score groups. **(F)** Differences in mRNAsi expression and correlation between high and low score groups. **(G)** Differences in expression and correlation between scores in high and low score groups and tumor mutational burden. **(H)** Differences in stemness between high and low score groups. **(I)** to compare the differences of tumor phenotypic indicators between high and low score groups. * p < 0.05, ** p < 0.01, *** p < 0.001.

To explore the molecular classification of patients based on our scoring system, we first performed correlation analysis between the score and all genes, identifying the top 50 genes that were positively and negatively correlated (**Figures 5A, B**). Following this correlation analysis, (**Figure 5C**) GSEA enrichment analysis revealed that the high-score group activated pathways associated with androgen response, hypoxia, and glycolysis, while inhibiting the unfolded protein response. (**Figure 5D**) Patients in the high-score group exhibited increased activation of various metabolic activities and a reduced response to drugs, which may be linked to drug resistance.

We also evaluated mDNAsi and mRNAsi, which are indicators related to cell stemness, reflecting changes in mRNA and DNA expression profiles associated with stemness. Results revealed that the high-score group exhibited a higher level of tumor stemness phenotype (**Figures 5E, F**) alongside an increased tumor mutational burden (TMB) (**Figure 5G**). Additionally, the stemness-related signature score was significantly elevated in the high-score group (**Figure 5H**). Moreover, ssGSEA was utilized for scoring, revealing that the high-score group was positively correlated with phenotypes that promote tumor progression, such as angiogenesis, apoptosis, cell cycle, and epithelial-mesenchymal transition (**Figure 5I**). Finally, the oncoPredict package was employed to predict drug sensitivity for patients in the high-score group who have poor prognosis, with lower IC50 values indicating higher sensitivity (**Supplementary Figure 1A**); for instance, docetaxel and lapatinib may be effective treatments for these high-score patients.

### 3.5 Functional exploration of PTGES3 in lung cancer

**Figure 6:**
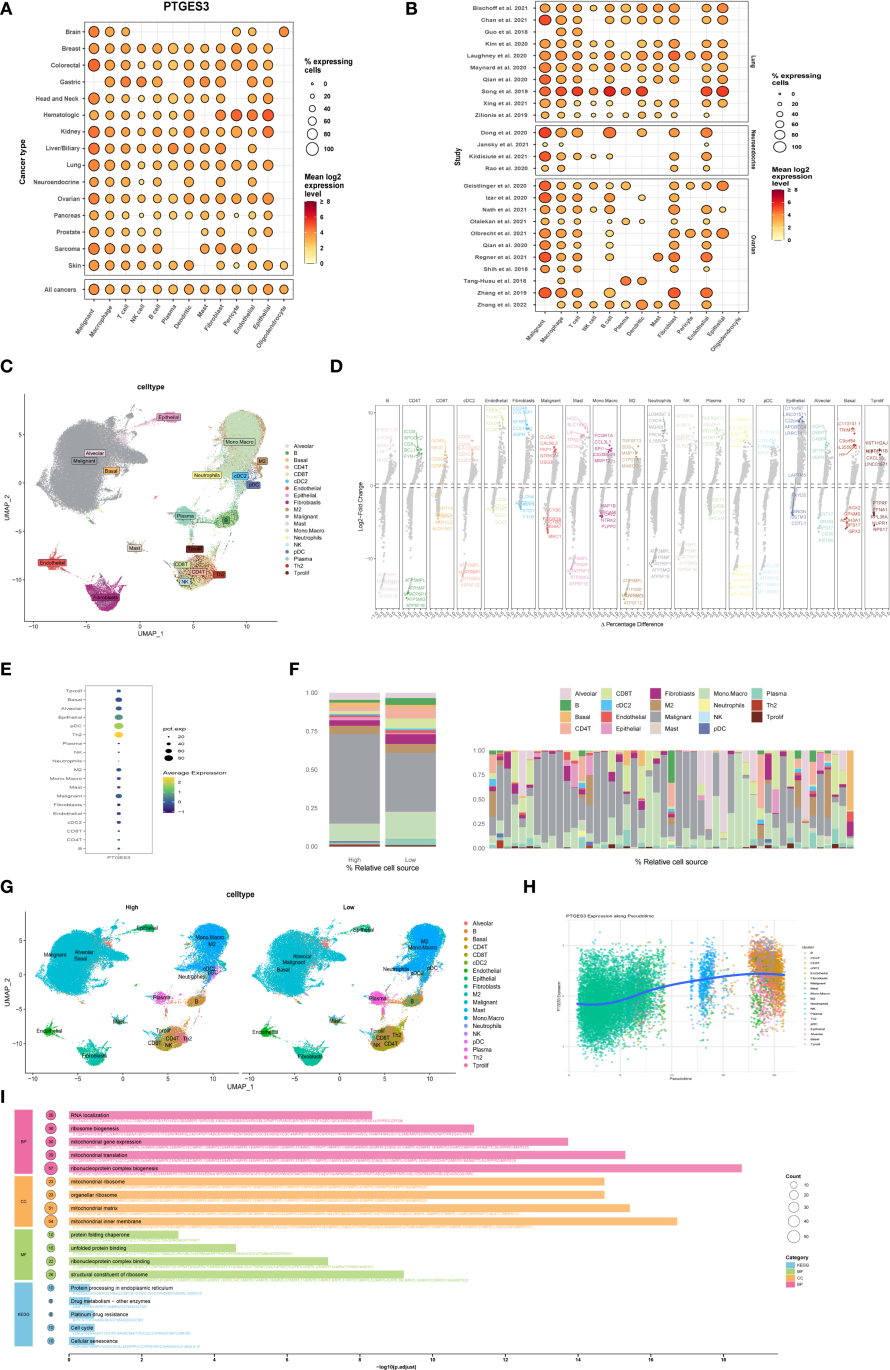
Functional exploration of PTGES3 in lung cancer. **(A)** Gene expression analysis across multiple tumor single-cell datasets, showing that PTGES3 is universally expressed in both tumor cells and the tumor microenvironment. **(B)** Gene expression profiles for each single-cell dataset. **(C)** Dimensionality reduction results of single-cell data, demonstrating cell clustering. **(D)** Identification of differentially expressed genes across various cell types using the FindAllMarkers function, highlighting the top five genes with high and low expression levels within each cell type. **(E)** Additional gene expression analysis in single-cell data. **(F)** Left panel shows high and low expression groups, while the right panel presents the proportion of each cell type for each patient. **(G)** UMAP visualizations of cell distribution in high and low expression groups. **(H)** Cell trajectory analysis conducted with the R package Slingshot, illustrating the relationship between gene expression and the proposed temporal sequence, indicating that more mature cells exhibit higher gene expression. **(I)** Enrichment analysis of differential genes between high and low expression groups based on single-cell data, performed using the R package clusterProfiler, with results presented separately for Biological Process (BP), Molecular Function (MF), Cellular Component (CC), and KEGG pathways.

PTGES3 plays a significant role in lung cancer. This study further investigates the function of PTGES3 in tumors. PTGES3 is ubiquitously expressed in various tumor cells and the tumor microenvironment (**Figure 6A**), demonstrating notably high expression levels in lung cancer studies (**Figure 6B**). Dimensionality reduction analysis of single-cell data revealed distinct cell clustering (**Figure 6C**) and highlighted the top five genes with both high and low expression in each cell type (**Figure 6D**), with PTGES3 showing high expression across multiple cell subsets (**Figure 6E**).

Furthermore, we categorized the samples into two groups based on the expression levels of PTGES3 to assess its impact on each subgroup. The results indicated significant changes in both tumor cells and macrophages with elevated PTGES3 expression, suggesting a potential close relationship between the two (**Figure 6F**). Additionally, UMAP maps illustrating cell distributions for the high and low expression groups are presented separately (**Figure 6G**). Cell trajectory analysis of PTGES3 expression revealed that higher levels of gene expression were associated with increased cell maturity (**Figure 6H**). Finally, enrichment analysis of the differentially expressed genes between the high and low expression groups demonstrated that elevated PTGES3 expression was closely associated with biological processes such as the cell cycle and endoplasmic reticulum stress (**Figure 6I**).

### 3.6 Cell-to-cell communication analysis reveals distinct communication pathways associated with high and low expression of PTGES3

**Figure 7:**
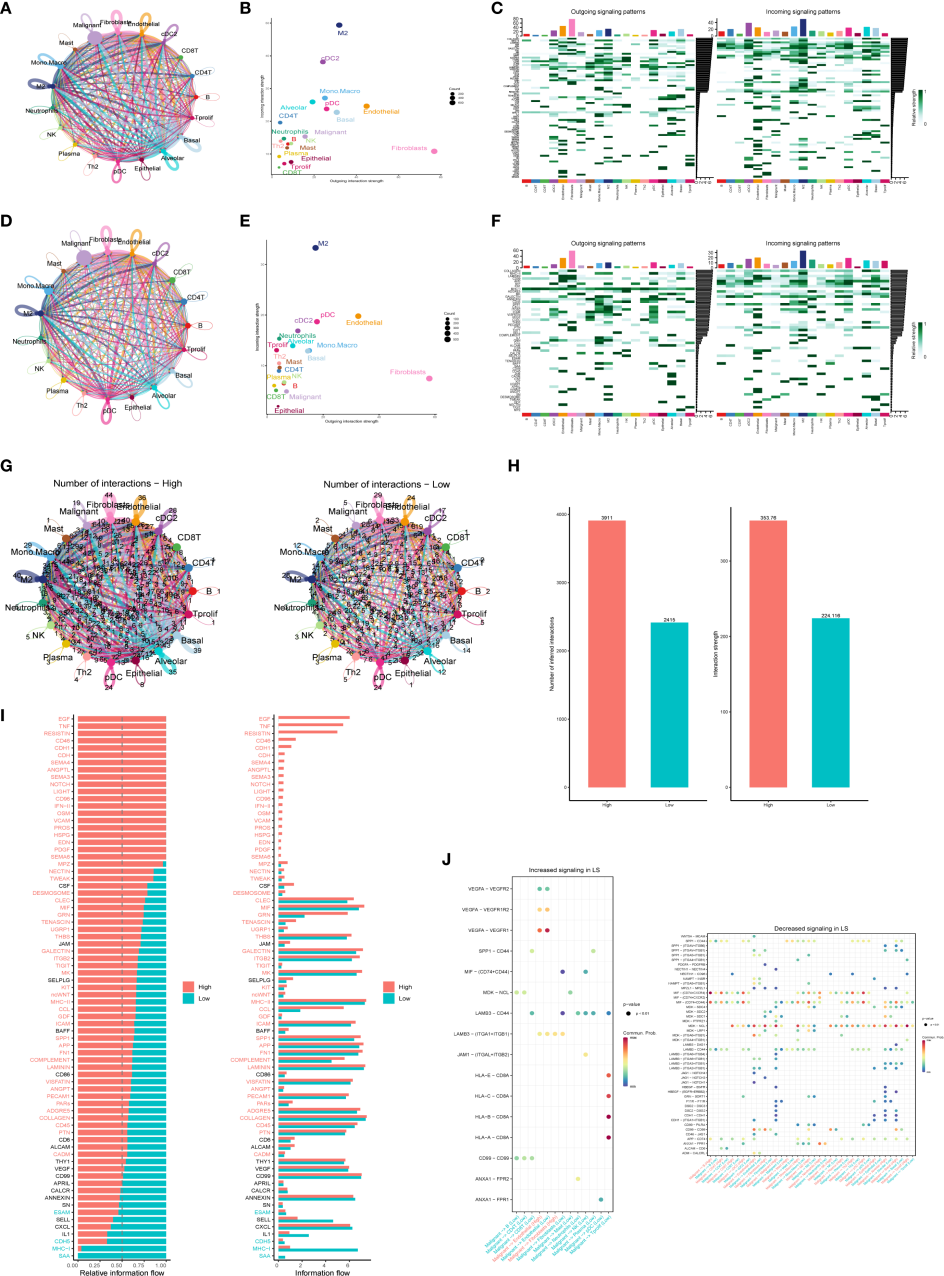
Intercellular communication analysis revealing specific pathways linked to high and low expression of PTGES3. **(A)** Circle diagram illustrating the number of interactions in the high expression group. **(B)** Scatter plots highlighting the distribution of efferent and afferent intensities in interactions (high and low), showing the strength of interactions between different cell types in the high expression group. **(C)** Heat maps displaying the output (left) and input (right) signal intensities of each signaling pathway in various cell types within the high expression group. **(D)** Number of interactions among cells in the low expression group. **(E)** Scatter plots depicting the distribution of efferent and afferent intensities in interactions (high and low), illustrating the strength of interactions between different cell types in the low expression group. **(F)** Heat maps showing the output (left) and input (right) signal intensities of each signaling pathway in different cell types within the low expression group. **(G)** Comparison of the number of interactions between different cell types in the high and low expression groups. **(H)** Bar graphs depicting the number and strength of interactions among various cell types. **(I)** Bar chart representing the two states of “High” (red) and “Low” (cyan) to visually display information flow related to different genes. **(J)** Bubble plots showcasing enhanced and attenuated ligand-receptor pairs in the low-score group.

Tumorigenesis is a complex and multistep process influenced by numerous factors. Recent studies have underscored the significance of intercellular communication in tumor evolution (21, 22). In this study, the R package “CellChat” was used to evaluate the interaction between cancer cells and immune cells in patients with high and low PTGES3 expression (LS score). We analyzed the number of cell interactions, interaction weights, and the input (right) and output (left) signal intensities of various signaling pathways (**Figures 7A-F**). The results indicated that the strength of interactions between patients with high LS scores and other cell subsets was significantly greater than that in patients with low scores, particularly with macrophages and fibroblasts (**Figures 7G, H**). This finding suggests that intercellular communication within the immune response plays a crucial role in facilitating immune cell infiltration in patients with high LS scores.

Additionally, cell-to-cell communication analysis unveiled specific communication pathways that exhibited high and low score specificity. The information flow graph generated by CellChat revealed a significant difference in signaling between the high-score group (red) and the low-score group (green). Specifically, the signaling pathways were notably more robust in the high-score group (indicated in red text), while signaling in the low-score group was more pronounced in green text; no significant differences in signaling were observed in the black text (**Figure 7I**). We further examined ligand-receptor interactions associated with enhanced LS signaling (**Figure 7J**). The results demonstrated that the interaction between VEGFA and its receptor was significantly heightened in the high PTGES3 expression state (P < 0.01), suggesting that the VEGF signaling pathway may promote tumor angiogenesis. Moreover, the interaction between SPP1 and CD44 highlights its potential role in facilitating cell adhesion and migration, which may contribute to tumor metastasis. The observed trend of increased interactions between HLA molecules and CD8+ T cells indicates a potential immunosuppressive mechanism, consistent with the results of tumor phenotype assessments. Overall, the enhanced signaling pathways and cellular interactions identified in this study provide a foundation for future functional studies and potential therapeutic targets.

### 3.7 PTGES3 upstream transcriptional regulation analysis

**Figure 8:**
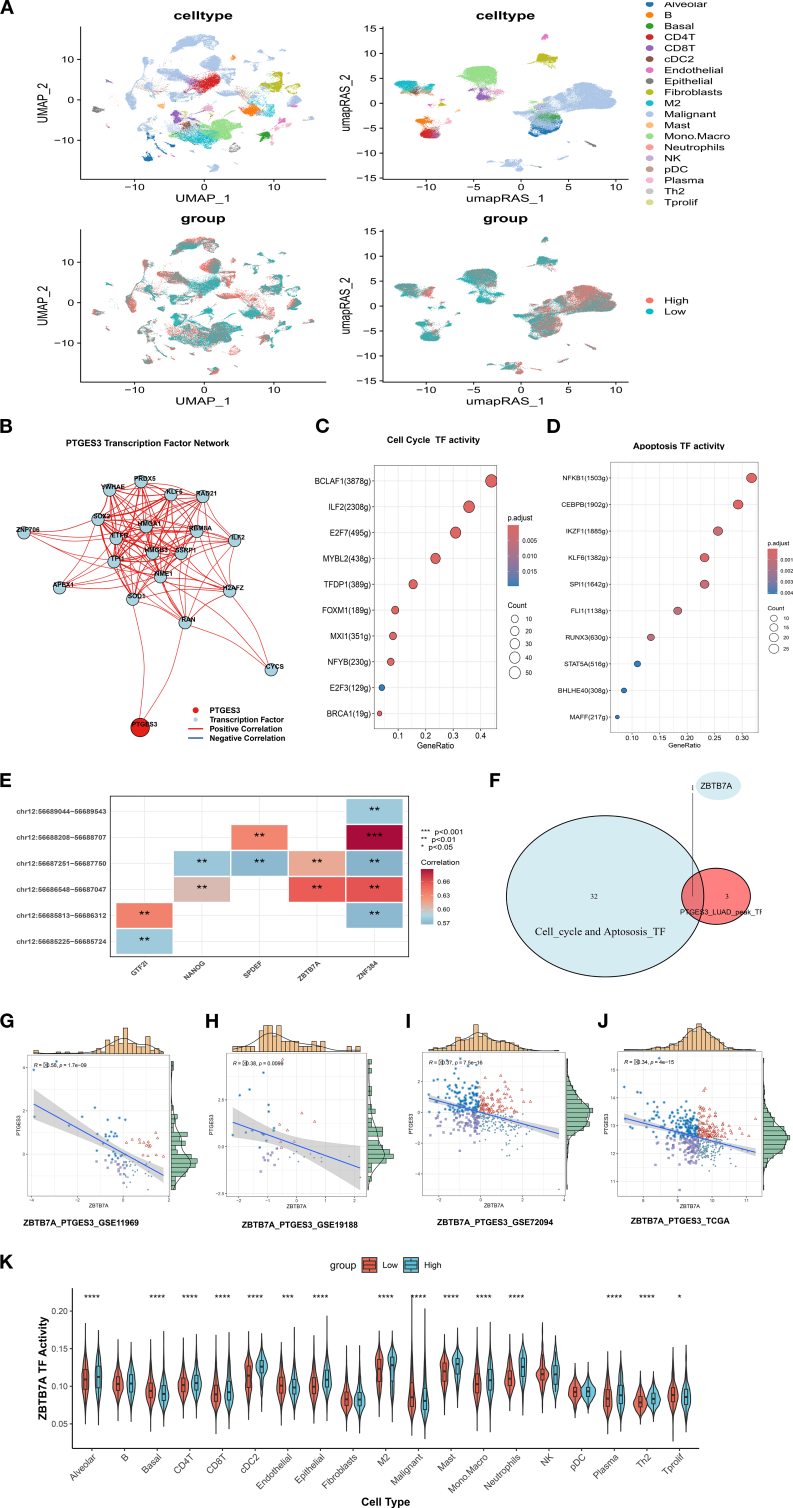
PTGES3 upstream transcriptional regulation analysis. **(A)** Expression of RAS-based single-cell UMAPs according to PTGES. **(B)** Prediction of transcription factors upstream of PTGES3 using pySCENIC data. **(C)** Transcription factors involved in regulating the cell cycle. **(D)** Transcription factors implicated in apoptosis regulation. **(E)** Heatmap showing the correlation between various transcription factors and ATAC peaks. **(F)** Venn diagram illustrating the intersection of transcription factors that regulate PTGES3 cell cycle and apoptosis, as predicted by Chip analysis. **(G)** Scatter plot of correlation between ZBTB7A and PTGES3 in GSE11969. **(H)** Scatter plot of correlation between GSE19188 ZBTB7A and PTGES3. **(I)** Scatter plot of correlation between ZBTB7A and PTGES3 in GSE72094. **(J)** Scatter diagram of correlation between ZBTB7A and PTGES3 in TCGA. **(K)** Activity of the ZBTB7A transcription factor varies between PTGES3 high and low expression groups within different cell subsets at the single-cell level. * p < 0.05, ** p < 0.01, *** p < 0.001, **** p < 0.0001.

We first employed the SCENIC method to identify key transcription factors. By reconstructing gene regulatory networks and analyzing single-cell RNA sequencing data, we identified significantly active transcription factors. SCENIC integrates cis-regulatory sequence information with RNA sequencing data, yielding transcription factor lists (including their enriched target gene sets) and transcription factor activity scores (RAS) for each cell. Utilizing the enhanced SCENIC method, we distinguished different cell types and PTGES3 expression levels based on transcription factor activity in UMAP maps (**Figure 8A**). Furthermore, using transcription factor data from pySCENIC, we predicted regulatory transcription factors upstream of PTGES3 (**Figure 8B**).

Previous studies indicate a close relationship between PTGES3 and the cell cycle (23). Consequently, we investigated transcription factors involved in regulating both the cell cycle and apoptosis based on the results of the improved SCENIC analysis (**Figures 8C, D**, **Supplementary Table 1**). Additionally, we analyzed the heatmap showing correlations between different transcription factors (TFs) and ATAC peaks in LUAD (**Figure 8E**). Combining these TFs with participants in the cell cycle and apoptosis pathways suggested that ZBTB7A may serve as a key regulatory TF (**Figure 8F**). Therefore, we further analyzed the correlation between them and found that there was a negative correlation between them in multiple data sets, so there may be a regulatory relationship between them (**Figure 8G-J**).Additionally, the activity of the ZBTB7A transcription factor varied significantly between PTGES3 high and low expression groups across different cell subsets (**Figure 8K**). Moreover, we confirmed the regulatory effects of the two by dual luciferase reporter assay (**Supplementary Figure 3G**).

To further explore the relationship between ZBTB7A and PTGES3, we examined the expression distribution of ZBTB7A across cell subpopulations after annotating the single-cell dataset NSCLC_GSE127465 (**Supplementary Figures 2A, B**). Building on this, we explored the expression level of ZBTB7A across pan-cancer types (**Supplementary Figure 2C**) and analyzed interactions between ZBTB7A+ and ZBTB7A- tumor cells (**Supplementary Figures 2D-G**). Remarkably, ZBTB7A+ tumor cells displayed stronger interactions with macrophages than with other cell types. Furthermore, the interaction between LGALS9 and its associated proteins (P4HB and CD44) with ZBTB7A was particularly significant, especially between LGALS9 and P4HB (**Supplementary Figures 2H-K**). As a key chaperone protein, P4HB is involved in protein folding and quality control during endoplasmic reticulum stress and is closely linked to ferroptosis. The interaction between P4HB and LGALS9 may significantly impact the survival, proliferation, and migration of tumor cells.

### 3.8 PTGES3 expression in tumors and mechanistic validation

**Figure 9:**
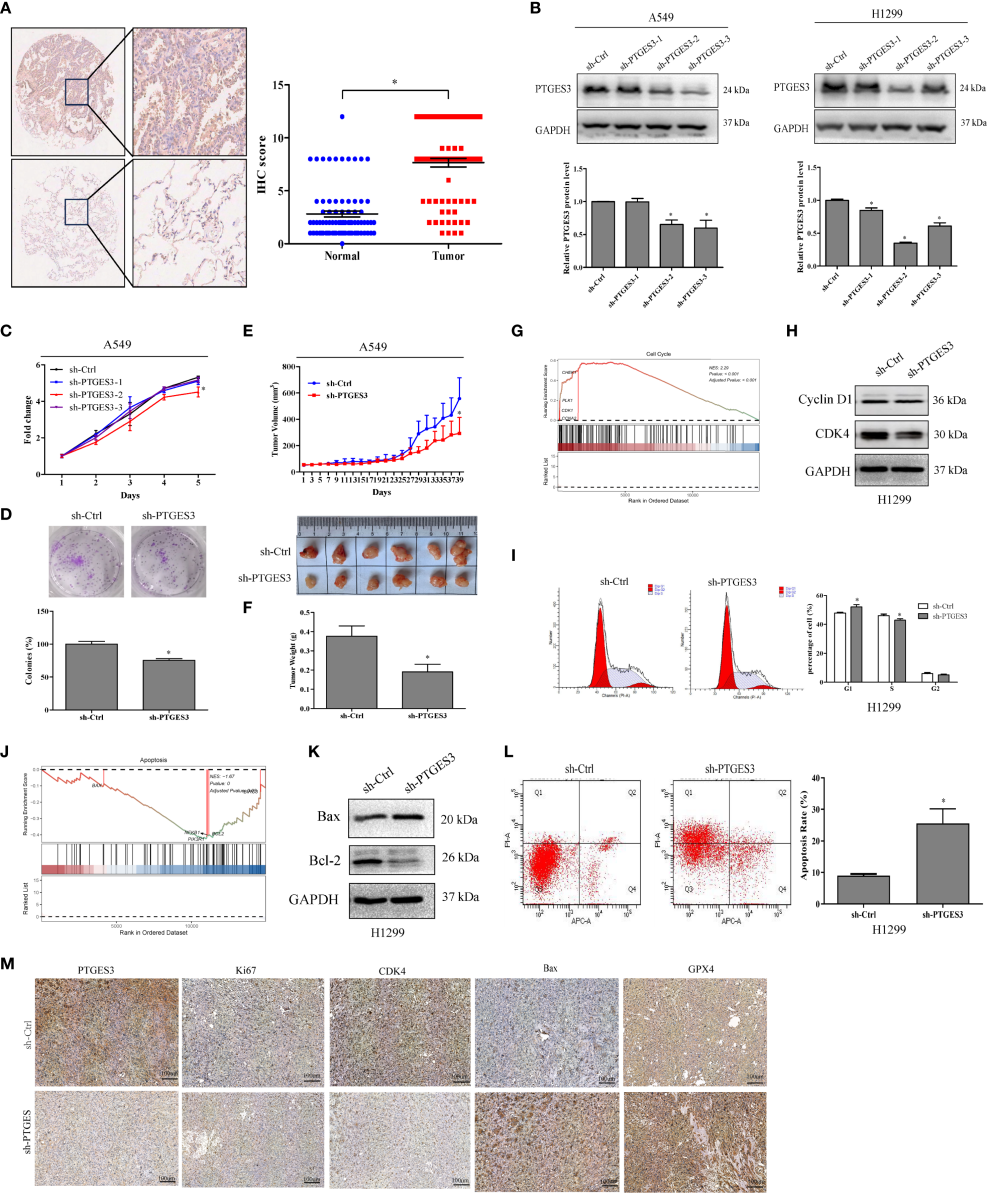
PTGES3 expression in tumors and mechanistic validation. **(A)** PTGES3 protein expression was assessed in lung cancer tissues and non-cancerous colorectal tissues via IHC. **(B)** Knockdown of PTGES3 in H1299 and A549 cells using three sh-PTGES3 sequences was verified by Western blot. **(C)** The effect of PTGES3 knockdown on lung cancer cell growth was evaluated with a CCK-8 assay, normalizing to Day 1 viability. **(D)** Colony formation analysis demonstrated reduced survival in cells with PTGES3 knockdown. **(E, F)** *In vivo* effects of PTGES3 knockdown on tumor growth were studied in a xenograft nude mouse model. **(G)** The tumor subset enrichment analysis using single-cell sequencing data indicated a GSEA enrichment analysis for samples with high PTGES3 expression. **(H)** Protein levels of Cyclin D1 and CDK4 were assessed in H1299 cells. **(I)** Flow cytometry analysis revealed cell cycle changes in H1299 cells following PTGES3 knockdown. **(J)** enrichment analysis of tumor subsets from single-cell sequencing data also suggested GSEA enrichment for samples with elevated PTGES3 expression. **(K)** The protein levels of Bax and Bcl-2 were evaluated through Western blot analysis. **(L)** Flow cytometry analysis demonstrated alterations in apoptosis in H1299 cells with PTGES3 knockdown. **(M)** The histochemical differences among various tumor groups for each index were illustrated. Statistical significance was indicated as P<0.05 for all relevant comparisons. * p < 0.05.

PTGES3 was found to be highly expressed in LUAD tissues (**Figure 9A**), and its expression was summarized alongside the clinicopathological features of lung cancer patients (**Supplementary Table 2**). Stable cell lines were constructed (**Figure 9B**), and CCK-8 and colony formation assays confirmed that the knockdown of PTGES3 inhibited cell proliferation (**Figures 9C, D**, **Supplementary Figures 3A, B**). These findings were further validated through animal experiments (**Figures 9E, F**, **Supplementary Figures 3C, D** **).** Enrichment analysis revealed a close association between PTGES3 and the cell cycle, which was further validated by Western blot analysis showing its impact on key cell cycle proteins (**Figures 9G, H**). Flow cytometry results demonstrated that knockdown of PTGES3 inhibited cell cycle progression (**Figure 9I**). Additionally, our investigations showed that PTGES3 also played a role in cell apoptosis, with Western blot analysis confirming its effect on apoptosis-related proteins (**Figures 9J, K**). Flow cytometry results indicated that PTGES3 knockdown increased the proportion of apoptotic cells (**Figure 9L**). Moreover, the histochemical results of the tumors further confirmed the above results at the protein level (**Figure 9M**).

Furthermore, we analyzed the pathway differences among various cell types in the positive and negative PTGES3 expression groups using single-cell RNA sequencing data from GSE127465 (**Supplementary Figure 3E**). We observed that high PTGES3 expression was closely associated with ferroptosis. We observed that high levels of PTGES3 expression were strongly associated with ferroptosis. Furthermore, cellular RNA sequencing data indicated that PTGES3 knockdown led to an enrichment of cell cycle pathways (**Supplementary Figure 3F**). Interaction analyses revealed that PTGES3+ tumor cells exhibited stronger direct interactions with macrophages compared to other cell types (**Supplementary Figures 4A-E**). Notably, the interactions between LGALS9 and its associated proteins (P4HB and CD44) with PTGES3+ also played a significant role, particularly the interaction between LGALS9 and P4HB, which was markedly stronger (**Supplementary Figures 4F-H**). Therefore, ZBTB7A may regulate the functions of macrophages and tumor cells via PTGES3. Additionally, the interplay among LGALS9, P4HB, and CD44 may enhance this regulatory effect. Through these signaling interactions, the cells can establish a complex network that significantly influences the immune status of the tumor microenvironment and tumor progression.

In our upstream genomic mutation analysis of PTGES3, we investigated the relationship between the ZBTB7A transcription factor and PTGES3. Our findings indicated that ZBTB7A was closely associated with mutations in TP53, CSMD3, KRAS, and ZFHX4 (**Supplementary Figure 5A**). Conversely, PTGES3 exhibited a strong correlation with mutations in CSMD3 and KEAP1 (**Supplementary Figure 5B**). Through an examination of several key mutations and their interrelatedness, we discovered that mutations in CSMD3 and KEAP1 significantly influenced their expression levels (**Supplementary Figures 5C, D**), while TP53 mutations were found to impact ZBTB7A expression. Importantly, we noted that these mutations did not exert a significant effect on PTGES3 expression (**Supplementary Figure 5E**). We further explored the implications of these mutations on tumor dynamics. Both CSMD3 mutations and PTGES3 exhibited similar effects on the cell cycle and the unfolded protein response (**Supplementary Figure 5F**), while KEAP1 mutations also influenced the cell cycle and a variety of metabolic activities (**Supplementary Figure 5G** **).** Therefore, in populations with CSMD3 mutations, the aberrant expression of ZBTB7A and PTGES3 appears to be associated with tumor progression.

## 4 Discussion

In our study, we identified 28 differentially expressed genes (DEGs) associated with prognosis and developed a set of consensus prognostic survival scores (LS scores) utilizing machine learning algorithms; this scoring system demonstrated strong predictive capability for overall survival at 1, 3, and 5 years (**Figure 2G**). Furthermore, analysis of the patient demographics in the high-score and low-score groups revealed that patients in the high-score group had a poorer prognosis (**Figure 2J**), which was closely linked to clinical progression indicators (**Figures 4A-E**). Notably, male patients tended to have higher scores, aligning with the findings of previous enrichment analyses. In addition, the prevalence of mutations in genes such as TP53, TTN, and MUC16 was significantly higher in the high-score group compared to the low-score group (**Figures 4F, G**), which corroborates prior studies indicating that TP53 mutations are strongly associated with poor prognosis in lung adenocarcinoma (24–26). Moreover, most of the differentially expressed genes between the two mutation groups were linked to tumor progression; for instance, NPAP1 is associated with potential molecular features, prognostic indicators, and possible therapeutic targets for neuroendocrine neoplasms (NEN) (27), while MTCL1 is closely related to the stemness of colorectal cancer and TRERF1 is associated with the progression of human breast cancer and resistance to tamoxifen (28, 29). Finally, in terms of treatment response, both docetaxel and lapatinib may exhibit sensitivity and efficacy in patients with high scores (**Supplementary Figure 1A**).

The two types of patients exhibited significant differences in tumor behavior. In the high-score group, tumor phenotypes displayed distinct characteristics, with positive correlations observed among various phenotypes related to tumor progression (**Figure 5I**), such as angiogenesis, apoptosis, cell cycle regulation, and epithelial-mesenchymal transition, all of which align with their poor prognosis. Notably, a comprehensive analysis of information flow revealed that multiple signaling pathways were significantly enhanced in the PTGES3 high expression group (**Figures 7I, J**). For instance, the pronounced interaction between VEGFA and its receptors, VEGFR1 and VEGFR2, indicates that VEGF signaling may facilitate tumor angiogenesis in patients with high survival scores, thereby supplying essential nutrients and oxygen for tumor growth (30). Furthermore, the significant interaction between SPP1 and CD44 suggests its pivotal role in cell adhesion and migration, subsequently promoting tumor cell metastasis (31). These findings underscore that PTGES3 could serve as a valuable predictor for treatment selection, providing a crucial basis for developing more effective treatment strategies, particularly in the management of patients with LUAD.

In the construction of the survival score (LS score), we performed an interpretable SHAP analysis, highlighting the significance of key genes including KRT6A, PERP, SEC61G, PTTG1, and PTGES3(**Figure 3A**). Among these, KRT6A exhibited the highest contribution to the model, with its increased expression closely associated with poor prognosis. Studies indicate a significant correlation between KRT6A expression and factors such as tumor lymph node metastasis, TNM staging, and smoking history in non-small cell lung cancer (NSCLC) patients (32). It is suggested that KRT6A may influence cancer biology by promoting stem cell-like features, with regulatory mechanisms of G6PD expression potentially involving LSD1 and MYC signaling pathways (33, 34). These findings provide new perspectives on KRT6A as a potential driver and prognostic biomarker in NSCLC. Additionally, research on LUAD has revealed that individuals carrying the PERP-428CC genotype face a higher risk of developing lung cancer, as its CAT/GR expression is downregulated through the PTEN/MDM2/p53 signaling pathway (35). Tumor cells with low PERP activity have demonstrated the potential of PERP overexpression to inhibit cancer cell growth and enhance sensitivity to chemotherapy (36). Furthermore, SEC61G shows promise in predicting response to targeted therapy and immunotherapy among LUAD patients and plays a critical role in brain metastases and immune evasion in NSCLC (37, 38). Silencing PTTG1 effectively inhibits cell proliferation and migration while promoting apoptosis (39, 40).

PTGES3 may initially exhibit a protective effect on prognosis; however, its increased expression later appears to correlate with adverse prognostic outcomes, mirroring the trends observed in expression changes and tumor staging. Through *in vitro* and *in vivo* experiments (**Figures 9C-F**), we validated that high PTGES3 expression is associated with enhanced tumor proliferation, inhibited apoptosis, and promoted cell cycle progression. Additionally, we explored potential upstream transcriptional regulators of PTGES3. By utilizing SCENIC in conjunction with ATAC-seq data, we identified ZBTB7A as a key regulatory transcription factor for PTGES3 (**Figure 8F**). ZBTB7A is a transcriptional repressor belonging to the POZ/BTB and Krüppel (POK) transcription factor family (41, 42). In LUAD, ZBTB7A expression is relatively low and correlates with poor prognosis, Notably, approximately 6% of ZBTB7A genes in LUAD exhibit mutations, including loss-of-function mutations (43). Furthermore, reduced expression of ZBTB7A can activate signaling pathways associated with tumor progression, such as AXL, PVT1, and ELK1, indicating a potential tumor suppressor function in LUAD (43). Consistent with our findings, both ZBTB7A and PTGES3 are influenced by the same mutation, CSMD3 (**Supplementary Figure 5C**), which also impacts the regulation of the cell cycle. Furthermore, numerous pieces of evidence indicate a negative correlation between ZBTB7A and PTGES3. In addition, the positive outcome of the dual luciferase reporter assay further reinforces regulatory relationship between these two factors.

Interestingly, HP1γ, a protein that recognizes dimethylated and trimethylated histone 3 at lysine 9 (H3K9me2/3), is one of the most frequently amplified and overexpressed histone methylation recognition proteins in human LUAD (43). Research indicates that HP1γ can downregulate transcriptional repression to modulate ZBTB7A expression, subsequently upregulating multiple oncogenic factors such as AXL, PVT1, and ELK1. This suggests that PTGES3 might serve as a critical downstream protein impacted by these changes, providing a theoretical basis for targeting patients with limited treatment options in LUAD.

Despite providing valuable insights into the clinical significance of LS score characteristics, our study recognizes several limitations. Firstly, the analyses primarily relied on retrospective data, highlighting the necessity for future research to validate the clinical relevance of our findings. Given the complexity of LUAD and its diverse histological phenotypes, comprehensive mechanistic investigations and clinical studies are essential for a deeper exploration of the score’s role across different LUAD subtypes. Moreover, although we validated the differential gene expression of PTGES3 between LUAD and precancerous samples using tissue microarrays, further verification at the protein level for other key genes remains to be conducted. While we confirmed the impact of PTGES3 on tumor growth through *in vitro* and *in vivo* experiments and explored its principal upstream regulatory factors, a more detailed investigation into specific mechanisms will be necessary in future studies. Additionally, our LS score model lacks validation through Phase III randomized controlled trials to support its decision-making capabilities. Therefore, conducting high-quality, well-powered, multicenter randomized controlled trials with adequate follow-up is a critical step for substantiating our results.

## 5 Conclusion

In conclusion, our findings suggest that LS score may serve as a valuable prognostic indicator for patients with lung adenocarcinoma (LUAD), with PTGES3 identified as a potential key therapeutic target. However, further research is needed to address the aforementioned limitations, thereby enhancing the validity and applicability of our results. Future studies will contribute to the verification of the clinical utility of LS score and PTGES3 across different LUAD subtypes and promote the development of more personalized treatment strategies.

## Data Availability

The original contributions presented in the study are included in the article/**Supplementary Material**. Further inquiries can be directed to the corresponding authors.

## References

[1] Sung H Ferlay J Siegel RL Laversanne M Soerjomataram I Jemal A. Global cancer statistics 2020: GLOBOCAN estimates of incidence and mortality worldwide for 36 cancers in 185 countries. CA Cancer J Clin. (2021) 71:209–49. doi: 10.3322/caac.21660, PMID: 33538338

[2] Lahiri A Maji A Potdar PD Singh N Parikh P Bisht B. Lung cancer immunotherapy: progress, pitfalls, and promises. Mol Cancer. (2023) 22:40. doi: 10.1186/s12943-023-01740-y, PMID: 36810079 PMC9942077

[3] Allemani C Matsuda T Di Carlo V Harewood R Matz M Nikšić M. Global surveillance of trends in cancer survival 2000-14 (CONCORD-3): analysis of individual records for 37 513 025 patients diagnosed with one of 18 cancers from 322 population-based registries in 71 countries. Lancet. (2018) 391:1023–75. doi: 10.1016/S0140-6736(17)33326-3, PMID: 29395269 PMC5879496

[4] Bade BC Dela Cruz CS. Lung Cancer 2020: Epidemiology, Etiology, and Prevention. Clin Chest Med. (2020) 41(1):1–24. doi: 10.1016/j.ccm.2019.10.001, PMID: 32008623

[5] Tan N Li Y Ying J Chen W. Histological transformation in lung adenocarcinoma: Insights of mechanisms and therapeutic windows. J Trans Internal Med. (2024) 12:452–65. doi: 10.1515/jtim-2024-0019, PMID: 39513032 PMC11538883

[6] Yin Q Ma H Dong Y Zhang S Wang J Liang J. The integration of multidisciplinary approaches revealed PTGES3 as a novel drug target for breast cancer treatment. J Transl Med. (2024) 22:84. doi: 10.1186/s12967-024-04899-0, PMID: 38245717 PMC10800054

[7] Zhang P Zhang H Tang J Ren Q Zhang J Chi H. The integrated single-cell analysis developed an immunogenic cell death signature to predict lung adenocarcinoma prognosis and immunotherapy. Aging (Albany NY). (2023) 15:10305–29. doi: 10.18632/aging.205077, PMID: 37796202 PMC10599752

[8] He S Su L Hu H Liu H Xiong J Gong X. Immunoregulatory functions and therapeutic potential of natural killer cell-derived extracellular vesicles in chronic diseases. Front Immunol. (2023) 14:1328094. doi: 10.3389/fimmu.2023.1328094, PMID: 38239346 PMC10795180

[9] Zhao J Wu L Cai G Ou D Liao K Yang J. Targeting PGE2 mediated senescent neuron improves tumor therapy. Neuro-oncology. (2025) 27(6):1491–1506. doi: 10.1093/neuonc/noaf045, PMID: 39963753 PMC12309717

[10] Jiang W Wei Q Xie H Wu D He H Lv X. Effect of PTGES3 on the prognosis and immune regulation in lung adenocarcinoma. Anal Cell Pathol (Amst). (2023) 2023:4522045. doi: 10.1155/2023/4522045, PMID: 37416927 PMC10322580

[11] Gao P Zou K Xiao L Zhou H Xu X Zeng Z. High expression of PTGES3 is an independent predictive poor prognostic biomarker and correlates with immune infiltrates in lung adenocarcinoma. Int Immunopharmacol. (2022) 110:108954. doi: 10.1016/j.intimp.2022.108954, PMID: 35820363

[12] Han Y Wang Y Dong X Sun D Liu Z Yue J. TISCH2: expanded datasets and new tools for single-cell transcriptome analyses of the tumor microenvironment. Nucleic Acids Res. (2023) 51:D1425–D31. doi: 10.1093/nar/gkac959, PMID: 36321662 PMC9825603

[13] Gavish A Tyler M Greenwald AC Hoefflin R Simkin D Tschernichovsky R. Hallmarks of transcriptional intratumour heterogeneity across a thousand tumours. Nature. (2023) 618:598–606. doi: 10.1038/s41586-023-06130-4, PMID: 37258682

[14] Tsherniak A Vazquez F Montgomery PG Weir BA Kryukov G Cowley GS. Defining a cancer dependency map. Cell. (2017) 170:564–76.e16. doi: 10.1016/j.cell.2017.06.010, PMID: 28753430 PMC5667678

[15] Liu C-J Hu F-F Xie G-Y Miao Y-R Li X-W Zeng Y. GSCA: an integrated platform for gene set cancer analysis at genomic, pharmacogenomic and immunogenomic levels. Brief Bioinform. (2023) 24. doi: 10.1093/bib/bbac558, PMID: 36549921

[16] Yuan H Yan M Zhang G Liu W Deng C Liao G. CancerSEA: a cancer single-cell state atlas. Nucleic Acids Res. (2019) 47:D900–D8. doi: 10.1093/nar/gky939, PMID: 30329142 PMC6324047

[17] Bravo González-Blas C De Winter S Hulselmans G Hecker N Matetovici I Christiaens V. SCENIC+: single-cell multiomic inference of enhancers and gene regulatory networks. Nat Methods. (2023) 20:1355–67. doi: 10.1038/s41592-023-01938-4, PMID: 37443338 PMC10482700

[18] Chen D Xu L Xing H Shen W Song Z Li H. Sangerbox 2: Enhanced functionalities and update for a comprehensive clinical bioinformatics data analysis platform. Imeta. (2024) 3:e238. doi: 10.1002/imt2.238, PMID: 39429873 PMC11487553

[19] Weng KQ Liu JY Li H She LL Qiu JL Qi H. Identification of Treg-related prognostic molecular subtypes and individualized characteristics in clear cell renal cell carcinoma through single-cell transcriptomes and bulk RNA sequencing. Int Immunopharmacol. (2024) 130:111746. doi: 10.1016/j.intimp.2024.111746, PMID: 38442575

[20] Jiang S Li H Zhang L Mu W Zhang Y Chen T. Generic Diagramming Platform (GDP): a comprehensive database of high-quality biomedical graphics. Nucleic Acids Res. (2025) 53:D1670–d6. doi: 10.1093/nar/gkae973, PMID: 39470721 PMC11701665

[21] Raredon MSB Adams TS Suhail Y Schupp JC Poli S Neumark N. Single-cell connectomic analysis of adult mammalian lungs. Sci Adv. (2019) 5:eaaw3851. doi: 10.1126/sciadv.aaw3851, PMID: 31840053 PMC6892628

[22] Sheikh BN Bondareva O Guhathakurta S Tsang TH Sikora K Aizarani N. Systematic identification of cell-cell communication networks in the developing brain. iScience. (2019) 21:273–87. doi: 10.1016/j.isci.2019.10.026, PMID: 31677479 PMC6838536

[23] Gu Y Chen G Du Y. Screening of prognosis-related genes in primary breast carcinoma using genomic expression data. J Comput Biol. (2020) 27:1030–40. doi: 10.1089/cmb.2019.0131, PMID: 31718274

[24] Xue D Lin H Lin L Wei Q Yang S Chen X. TTN/TP53 mutation might act as the predictor for chemotherapy response in lung adenocarcinoma and lung squamous carcinoma patients. Transl Cancer Res. (2021) 10:1284–94. doi: 10.21037/tcr-20-2568, PMID: 35116455 PMC8798240

[25] Pelosi G. KEAP1 and TP53 (Co)mutation in lung adenocarcinoma: another bullet for immunotherapy? J Thorac Oncol. (2021) 16:1979–83., PMID: 34809800 10.1016/j.jtho.2021.10.004

[26] Vokes NI Chambers E Nguyen T Coolidge A Lydon CA Le X. Concurrent TP53 mutations facilitate resistance evolution in EGFR-mutant lung adenocarcinoma. J Thorac Oncol. (2022) 17:779–92. doi: 10.1016/j.jtho.2022.02.011, PMID: 35331964 PMC10478031

[27] Liu M Li N Tang H Chen L Liu X Wang Y. The mutational, prognostic, and therapeutic landscape of neuroendocrine neoplasms. Oncologist. (2023) 28:e723–e36. doi: 10.1093/oncolo/oyad093, PMID: 37086484 PMC10485279

[28] Geng S Zhu L Wang Y Liu Q Yu C Shi S. Co-Colorectal cancer stem cells employ the FADS1/DDA axis to evade NK cell-mediated immunosuppression after co-cultured with NK cells under hypoxia. Int Immunopharmacol. (2024) 143:113535. doi: 10.1016/j.intimp.2024.113535, PMID: 39488917

[29] van Agthoven T Sieuwerts AM Meijer-van Gelder ME Look MP Smid M Veldscholte J. Relevance of breast cancer antiestrogen resistance genes in human breast cancer progression and tamoxifen resistance. J Clin Oncol. (2009) 27:542–9. doi: 10.1200/JCO.2008.17.1462, PMID: 19075277

[30] Kaplan RN Riba RD Zacharoulis S Bramley AH Vincent L Costa C. VEGFR1-positive haematopoietic bone marrow progenitors initiate the pre-metastatic niche. Nature. (2005) 438:820–7. doi: 10.1038/nature04186, PMID: 16341007 PMC2945882

[31] Nallasamy P Nimmakayala RK Karmakar S Leon F Seshacharyulu P Lakshmanan I. Pancreatic tumor microenvironment factor promotes cancer stemness via SPP1-CD44 axis. Gastroenterology. (2021) 161. doi: 10.1053/j.gastro.2021.08.023, PMID: 34418441 PMC10069715

[32] Yang B Zhang W Zhang M Wang X Peng S Zhang R. KRT6A promotes EMT and cancer stem cell transformation in lung adenocarcinoma. Technol Cancer Res Treat. (2020) 19:1533033820921248. doi: 10.1177/1533033820921248, PMID: 32329414 PMC7225834

[33] Xu Q Yu Z Mei Q Shi K Shen J Gao G. Keratin 6A (KRT6A) promotes radioresistance, invasion, and metastasis in lung cancer via p53 signaling pathway. Aging (Albany NY). (2024) 16:7060–72. doi: 10.18632/aging.205742, PMID: 38656878 PMC11087103

[34] Zhou J Jiang G Xu E Zhou J Liu L Yang Q. Identification of SRXN1 and KRT6A as key genes in smoking-related non-small-cell lung cancer through bioinformatics and functional analyses. Front Oncol. (2021) 11:810301. doi: 10.3389/fonc.2021.810301, PMID: 35071014 PMC8767109

[35] Liao C-Y Yang S-F Wu T-J Chang H Huang C-YF Liu Y-F. Novel function of PERP-428 variants impacts lung cancer risk through the differential regulation of PTEN/MDM2/p53-mediated antioxidant activity. Free Radic Biol Med. (2021) 167:307–20. doi: 10.1016/j.freeradbiomed.2021.02.017, PMID: 33731308

[36] Shan B-Q Wang X-M Zheng L Han Y Gao J Lv M-D. DCAF13 promotes breast cancer cell proliferation by ubiquitin inhibiting PERP expression. Cancer Sci. (2022) 113:1587–600. doi: 10.1111/cas.15300, PMID: 35178836 PMC9128170

[37] Zhou C Yang Y Cui H Li S Wang Z Chen L. SEC61G facilitates brain metastases via antagonizing PGAM1 ubiquitination and immune microenvironment remodeling in non-small cell lung cancer. Int J Biol Sci. (2025) 21:1436–58. doi: 10.7150/ijbs.109187, PMID: 39990664 PMC11844280

[38] Chi H Huang J Yan Y Jiang C Zhang S Chen H. Unraveling the role of disulfidptosis-related LncRNAs in colon cancer: a prognostic indicator for immunotherapy response, chemotherapy sensitivity, and insights into cell death mechanisms. Front Mol Biosci. (2023) 10:1254232. doi: 10.3389/fmolb.2023.1254232, PMID: 37916187 PMC10617599

[39] Wang Z Zhang J Zuo C Chen H Wang L Xie Y. Identification and validation of tryptophan-related gene signatures to predict prognosis and immunotherapy response in lung adenocarcinoma reveals a critical role for PTTG1. Front Immunol. (2024) 15:1386427. doi: 10.3389/fimmu.2024.1386427, PMID: 39144144 PMC11321965

[40] Zhou Q Li L Sha F Lei Y Tian X Chen L. PTTG1 reprograms asparagine metabolism to promote hepatocellular carcinoma progression. Cancer Res. (2023) 83:2372–86. doi: 10.1158/0008-5472.CAN-22-3561, PMID: 37159932

[41] Liu X-S Haines JE Mehanna EK Genet MD Ben-Sahra I Asara JM. ZBTB7A acts as a tumor suppressor through the transcriptional repression of glycolysis. Genes Dev. (2014) 28:1917–28. doi: 10.1101/gad.245910.114, PMID: 25184678 PMC4197949

[42] Gupta S Singh AK Prajapati KS Kushwaha PP Shuaib M Kumar S. Emerging role of ZBTB7A as an oncogenic driver and transcriptional repressor. Cancer Lett. (2020) 483:22–34. doi: 10.1016/j.canlet.2020.04.015, PMID: 32348807

[43] Alam H Li N Dhar SS Wu SJ Lv J Chen K. HP1γ Promotes lung adenocarcinoma by downregulating the transcription-repressive regulators NCOR2 and ZBTB7A. Cancer Res. (2018) 78:3834–48. doi: 10.1158/0008-5472.CAN-17-3571, PMID: 29764865 PMC6443096

