# Supplementary material for: Machine learning integration with multi-omics data constructs a robust prognostic model and identifies PTGES3 as a therapeutic target for precision oncology in lung adenocarcinoma
Source: Front Immunol. 2025 Sep 29;16:1651270. doi: 10.3389/fimmu.2025.1651270 (PMC12515886; doi:10.3389/fimmu.2025.1651270)
Supplement: Supplementary file 1 [file DataSheet1.docx]

Supplementary Material

# Supplementary Material

Supplementary table01. PTGES3-related TF regulatory list

Supplementary table02. Tissue microarray information

# Supplementary Figures and Tables

## Supplementary Figures

**
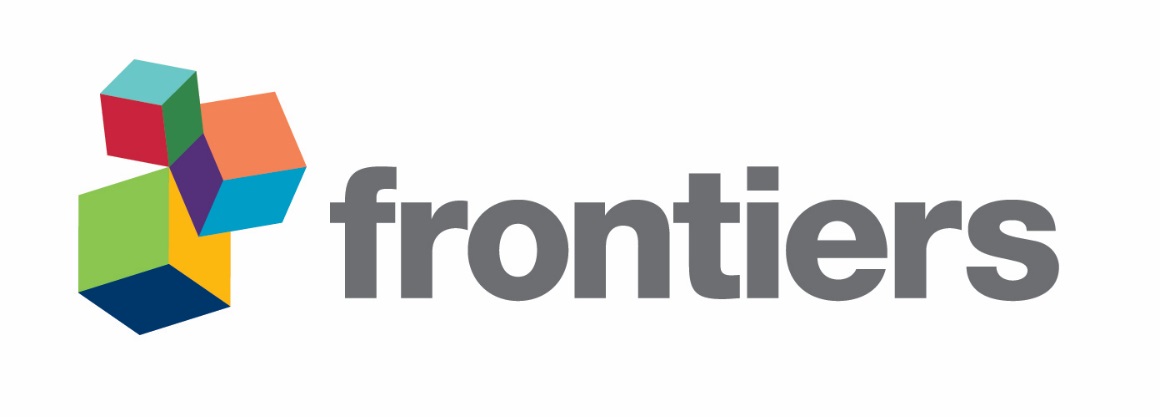
**


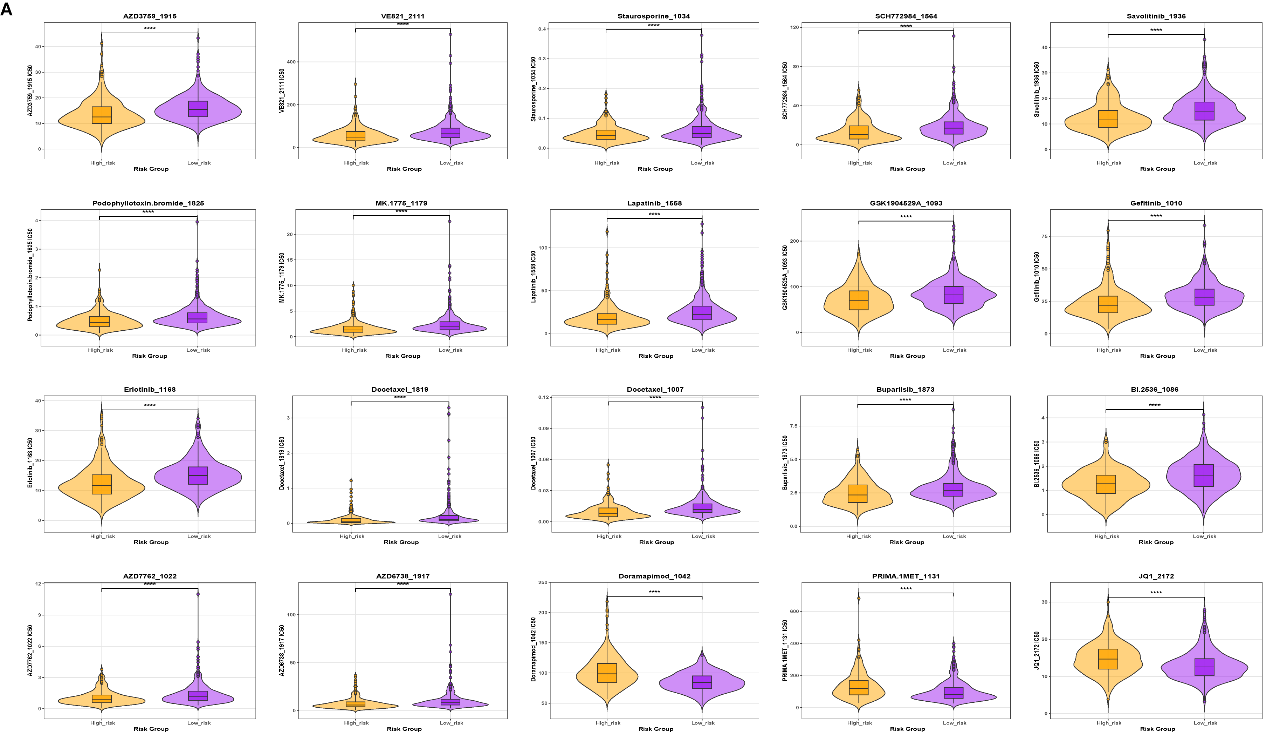


**Supplementary Figure 1. Drug Sensitivity Analysis in Patients with High and Low Scores.** (Supplementary Figure 1A) The oncoPredict package in R was utilized to predict the IC50 values for each sample across a range of anticancer drugs, enabling a comparison of differences between the high and low score groups. A higher IC50 value indicates lower sensitivity to treatment (the graphs display the top 20 drugs).


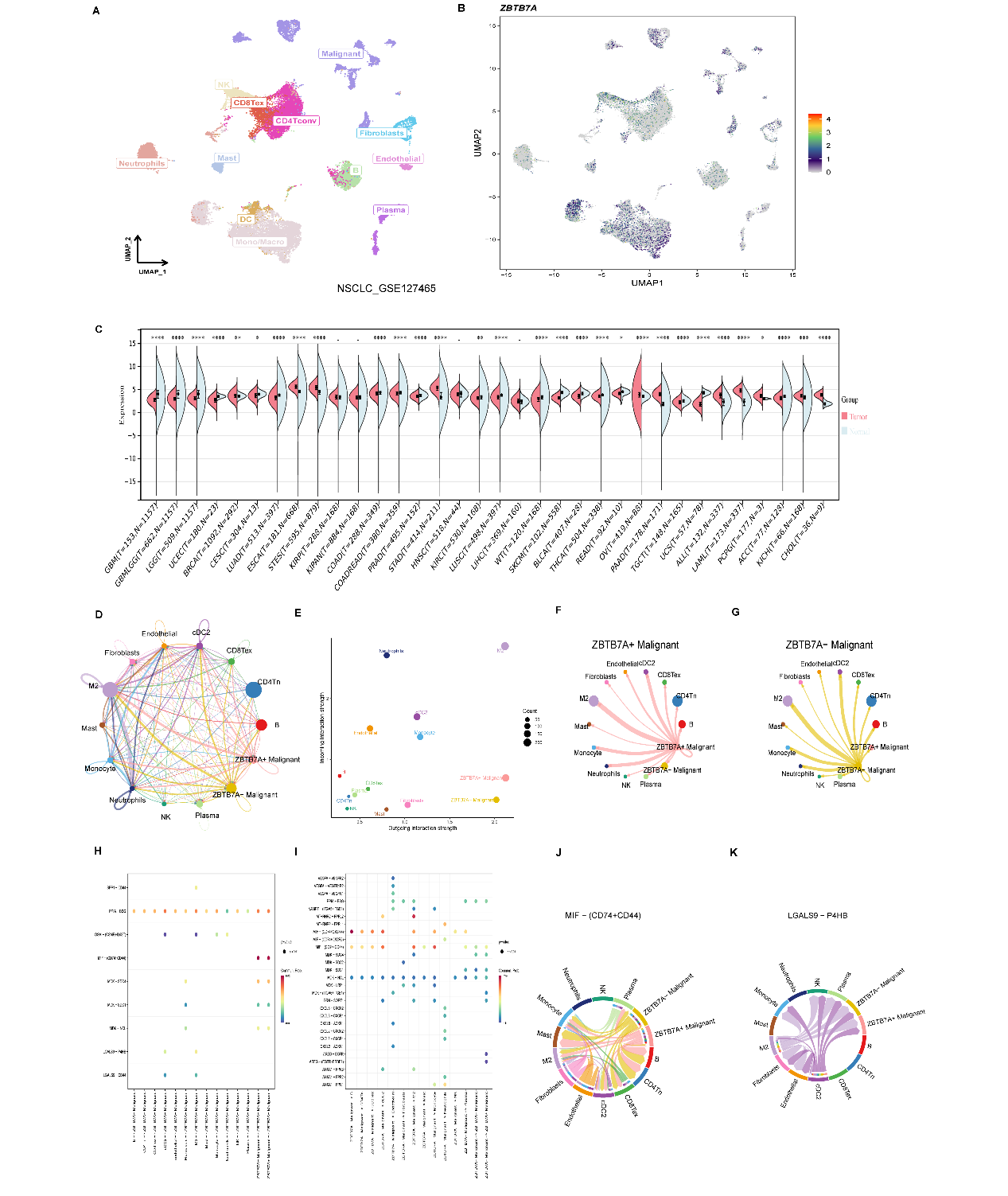


**Supplementary Figure 2. Exploring the relationship between ZBTB7A and PTGES3**. (Supplementary Figure 2A) Annotated UMAP map of the single-cell NSCLC_GSE127465 subgroup. (Figure 2B) Expression distribution of ZBTB7A in each subgroup of single-cell NSCLC_GSE127465. (Supplementary Figure 2C) Differential expression levels of ZBTB7A in pan-cancer. (Supplementary Figure 2D) Diagram of the interaction network between ZBTB7A+ and ZBTB7A− malignant cells and other cell types in NSCLC_GSE127465. (Supplementary Figure 2E) Interaction strength plots for each cell type. (Supplementary Figure 2F) Interaction network between ZBTB7A+ malignant cells and other cell types. (Supplementary Figure 2G) Interaction network between ZBTB7A− malignant cells and immune cells. (Supplementary Figure 2H) Results of significance analysis of cell-to-cell interactions, represented as a bubble plot showing interactions between different cell types (e.g., CD4+ T cells, B cells, monocytes) and the expression of tumor-related component ZBTB7A. (Supplementary Figure 2I) Detailed significance analysis plots of cell-to-cell interactions, with a bubble plot illustrating interactions between ZBTB7A+ malignant tumor cells and other cell types (e.g., CD4+ T cells, monocytes). (Supplementary Figure 2J) Ring heat map showing intercellular interactions between MIF and CD74_CD44, and the relationship between cell types (e.g., CD4+ T cells, B cells, monocytes) and the expression status of ZBTB7A. (Supplementary Figure 2K) Ring heat map demonstrating cell-to-cell interactions between LGALS9 and P4HB.


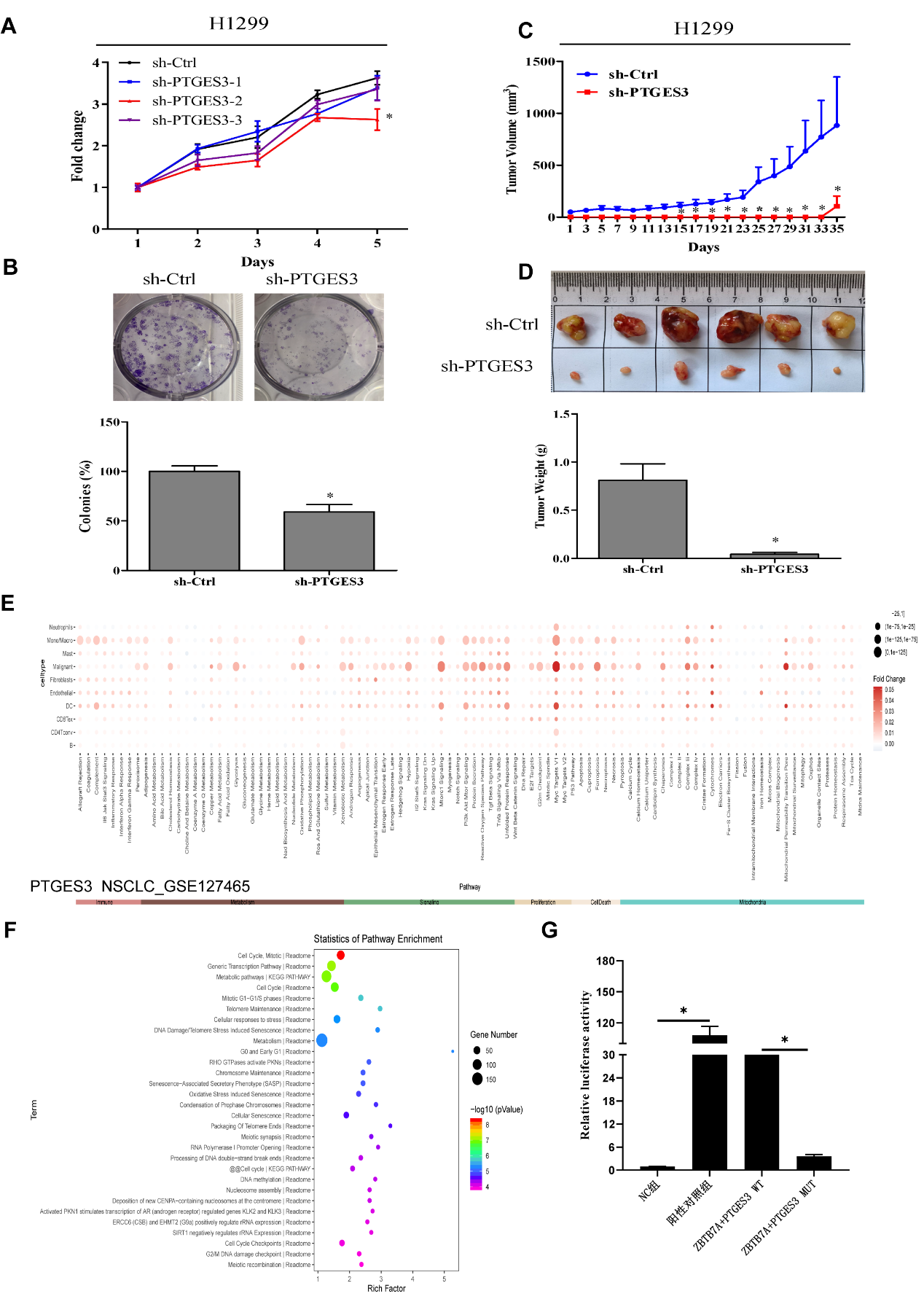


**Supplementary Figure 3. Functional exploration of PTGES3.** (Supplementary Figure 3A) PTGES3 knockdown on lung cancer cell growth was evaluated with a CCK-8 assay, normalizing to Day 1 viability. (Supplementary Figure 3B) Colony formation analysis demonstrated reduced survival in cells with PTGES3 knockdown. (Supplementary Figure 3C-D) In vivo effects of PTGES3 knockdown on tumor growth were studied in a xenograft nude mouse model**.** (Supplementary Figure 3E) Pathway differences between PTGES3 positive and negative groups were analyzed**.** (Supplementary Figure 3F) KEGG enrichment analysis of PTGES3 knockdown**.** (Supplementary Figure 3G) ZBTB7A and PTGES3WT and MUT dual luciferase reporter experiments**.**


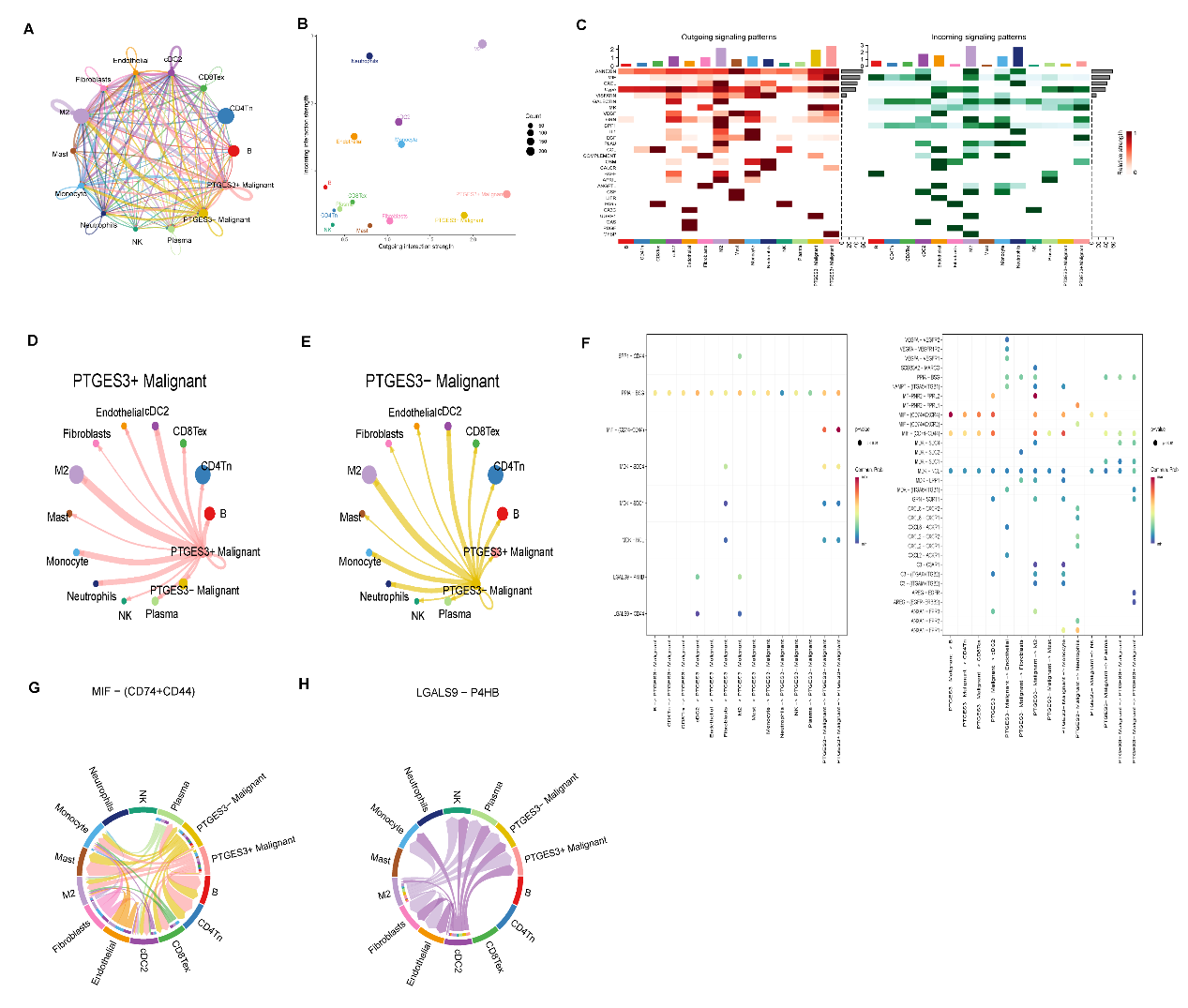
**Supplementary Figure 4. Interaction network between PTGES3 and LGALS9 regulated by ZBTB7A in the tumor microenvironment.** (Supplementary Figure 4A) Diagram of the interaction network between PTGES3+ and PTGES3− malignant cells and other cell types in NSCLC_GSE127465. (Supplementary Figure 4B) Interaction strength plots for each cell type. (Supplementary Figure 4C) Comparison of cell signaling patterns for each cell subpopulation, with the left part showing "Outgoing signaling patterns" and the right part displaying "Incoming signaling patterns." (Supplementary Figure 4D) Interaction network between PTGES3+ malignant cells and other cell types. (Supplementary Figure 4E) Interaction network between PTGES3− malignant cells and immune cells. (Supplementary Figure 4F) Results of significance analysis of cell-to-cell interactions, presented as a bubble plot demonstrating interactions between different cell types, such as CD4+ T cells, B cells, and monocytes, and the expression of PTGES3+ malignant tumor cells. (Supplementary Figure 4G) Ring heat map illustrating intercellular interactions between MIF and CD74_CD44, depicting the relationship among various cell types (e.g., CD4+ T cells, B cells, monocytes) and PTGES3 expression status. (Supplementary Figure 4H) Ring heat map demonstrating the cell-to-cell interaction between LGALS9 and P4HB.


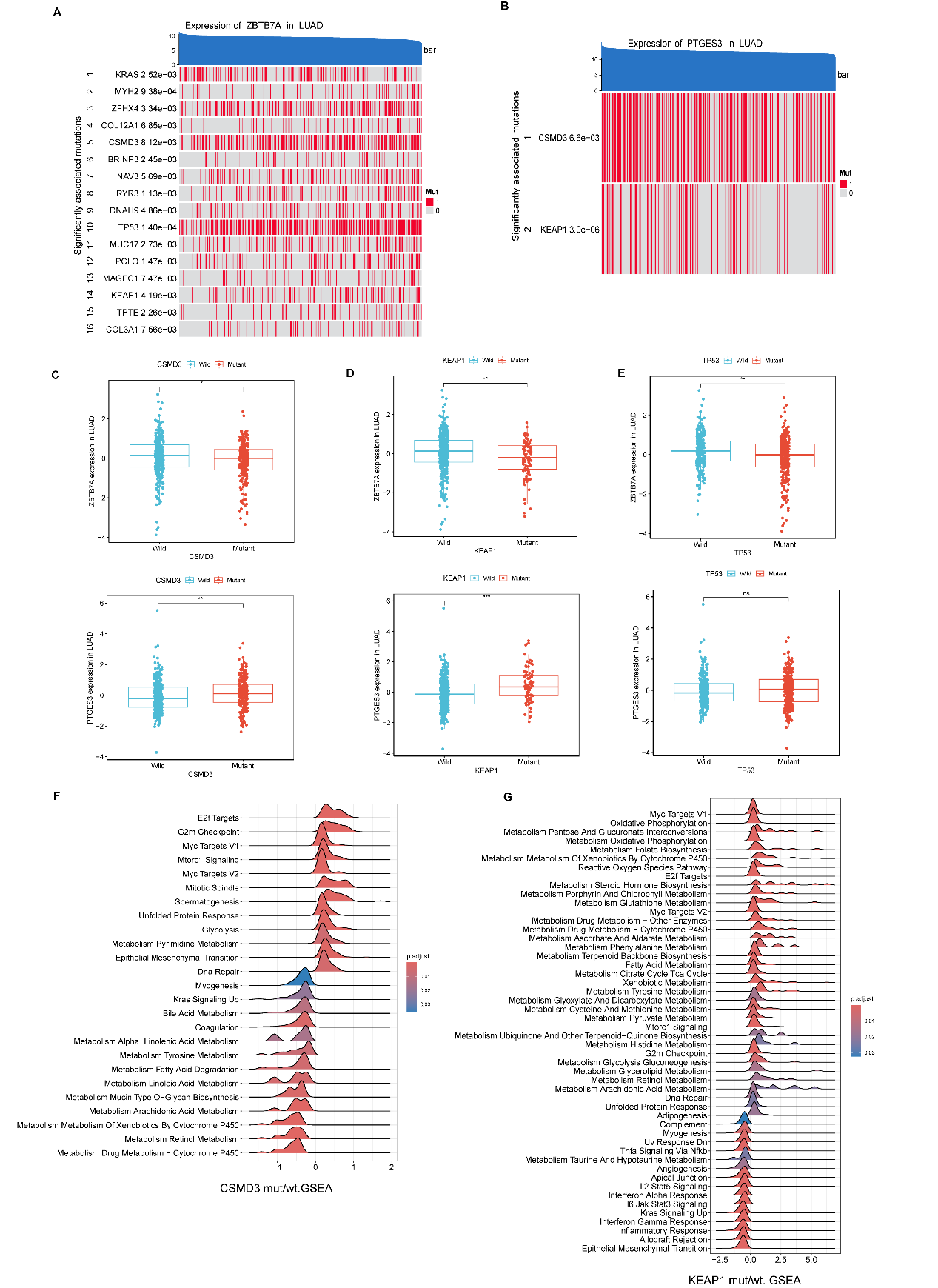


**Supplementary Figure 5. Comparative Analysis of Genetic Mutations in PTGES3 and ZBTB7A.** (Supplementary Figure 5A) Heat map of the relationship between ZBTB7A gene expression and mutation type in LUAD. (Supplementary Figure 5B) Heat map of the relationship between PTGES gene expression and mutation type in LUAD.(Supplementary Figures 5C) ZBTB7A and PTGES gene expression levels were significantly different between CSMD3 wild type and mutant type.(Supplementary Figures 5D) The expression levels of ZBTB7A and PTGES genes were significantly different between KEAP1 wild type and mutant type.(Supplementary Figures 5E) ZBTB7A and PTGES gene expression levels were significantly different between TP53 wild-type and mutant types.(Supplementary Figures 5F) Pathway enrichment analysis in patients with CSMD3 mutation.(Supplementary Figures 5G) pathway enrichment analysis of KEAP1 mutant patients.
